# Prp8 regulates oncogene-induced hyperplastic growth in *Drosophila*

**DOI:** 10.1242/dev.162156

**Published:** 2018-11-12

**Authors:** Cecilia H. Fernández-Espartero, Alberto Rizzo, Alexander D. Fulford, Julia Falo-Sanjuan, Damien Goutte-Gattat, Paulo S. Ribeiro

**Affiliations:** 1 Centre for Tumour Biology, Barts Cancer Institute, Queen Mary University of London, Charterhouse Square, London EC1M 6BQ, UK; 2 Department of Physiology, Development and Neuroscience, University of Cambridge, Downing Street, Cambridge CB2 3DY, UK

**Keywords:** Prp8, Tumour growth, *Drosophila*, Spliceosome, Ras

## Abstract

Although developmental signalling pathways control tumourigenic growth, the cellular mechanisms that abnormally proliferating cells rely on are still largely unknown. *Drosophila melanogaster* is a genetically tractable model that is used to study how specific genetic changes confer advantageous tumourigenic traits. Despite recent efforts, the role of deubiquitylating enzymes in cancer is particularly understudied. We performed a *Drosophila in vivo* RNAi screen to identify deubiquitylating enzymes that modulate Ras^V12^-induced hyperplastic growth. We identified the spliceosome core component Prp8 as a crucial regulator of Ras-, EGFR-, Notch- or RET-driven hyperplasia. Loss of *prp8* function alone decreased cell proliferation, increased cell death, and affected cell differentiation and polarity. In hyperplasia, Prp8 supported tissue overgrowth independently of caspase-dependent cell death. The depletion of *prp8* efficiently blocked Ras-, EGFR- and Notch-driven tumours but, in contrast, enhanced tumours that were driven by oncogenic RET, suggesting a context-specific role in hyperplasia. These data show, for the first time, that Prp8 regulates hyperplasia, and extend recent observations on the potential role of the spliceosome in cancer. Our findings suggest that targeting Prp8 could be beneficial in specific tumour types.

## Abstract (precis)

**Summary:** Prp8 has been identified as a modulator of oncogenic growth in multiple *Drosophila* cancer models, which suggests the spliceosome as a potential context-dependent target in cancers.

## INTRODUCTION

Intensive research in the recent past that has combined molecular profiling approaches with *in vivo* and *in vitro* functional studies has resulted in the identification of genes and pathways that drive tumour formation (Hanahan and Weinberg, 2011). In this regard, the use of the fruit fly *Drosophila melanogaster* as a model organism has been particularly powerful (Gonzalez, 2013; Sonoshita and Cagan, 2017; Tipping and Perrimon, 2014). Indeed, seminal studies using *Drosophila* have led to the identification of multiple genes and signalling pathways, including the Notch (N) and Ras/MAPK pathways that, when mutated, not only cause severe developmental defects but are also involved in tumourigenesis (Gonzalez, 2013). Indeed, different aspects of tumourigenesis have been studied in *Drosophila* and the vast majority of cancer hallmarks are conserved in flies (Hanahan and Weinberg, 2011; Tipping and Perrimon, 2014).

Signalling pathways underpin cellular behaviour and, when disrupted, lead to developmental defects and/or cellular transformation. Virtually all signalling pathways are controlled by post-translational protein modifications, with phosphorylation being the most frequently associated with signalling events (Hynes et al., 2013). However, it is clear that additional post-translational modifications are vital for tightly controlling developmental events. Ubiquitylation, a multi-step cascade that results in the covalent attachment of the small protein ubiquitin onto a substrate, has emerged as a crucial process in signalling that regulates virtually all functions within a cell (Heride et al., 2014). Despite being historically linked with regulation of protein levels and protein degradation, ubiquitylation can also have non-proteolytic effects, leading to changes in protein-protein interactions, protein function and subcellular localisation (Rape, 2017). In a manner akin to phosphorylation, ubiquitylation is reversible, and the removal of ubiquitin moieties from target proteins is controlled by deubiquitylating enzymes (DUBs) (Heride et al., 2014; Rape, 2017). However, the *in vivo* role of DUBs remains poorly explored. This is especially true in the context of developmental and oncogenic growth, despite the fact that many DUBs have recently been linked with tumourigenesis (Fraile et al., 2012).

We performed a *Drosophila in vivo* screening approach to study the role of genes containing domains that are involved in the removal of ubiquitin and ubiquitin-like proteins in the regulation of tumourigenesis. Our top hit was the spliceosome component Prp8, which we identified as a crucial regulator of developmental and hyperplastic growth in several *Drosophila* models of cancer. Prp8 is a core protein of the spliceosome complex and its protein structure includes an MPN/JAB domain typical of the JAMM family of DUBs (Grainger and Beggs, 2005; Komander et al., 2009). Based on sequence and structural analysis, Prp8 is thought to be an inactive DUB, as conserved residues of the JAMM ubiquitin hydrolase domain are absent (Clague et al., 2013; Pena et al., 2007). Nevertheless, the MPN/JAB domain is essential for Prp8 function and can bind ubiquitin with an affinity comparable with that of other ubiquitin-binding domains (Bellare et al., 2006). Our data suggest that Prp8 regulates hyperplasia in a context-dependent manner, which is consistent with previous observations that identified *prp8* as a regulator of organ growth *in vivo*, in a genetic modifier screen that used overexpression of a kinase-dead phosphoinositide 3-kinase (Coelho et al., 2005).

Together with recently published data, our work identifies the spliceosome as a potential target in cancers and suggests that tumours display different sensitivity to disruption of Prp8 function depending on the driver oncogene (Hsu et al., 2015). Thus, our results imply that future therapies that target the spliceosome in cancer may require the identification of the exact context-dependent condition of individual tumours to maximise their efficacy.

## RESULTS

### *In vivo* RNAi screening identifies Prp8 as a novel regulator of developmental and oncogene-induced growth

To elucidate the role of DUBs in the regulation of developmental and pathological growth, we performed *in vivo* RNAi screens using lines targeting all *Drosophila* genes that carry a ubiquitin hydrolase domain (Broemer et al., 2010). To explore the role of ubiquitin-related modifications, we also included *Drosophila* orthologues of SUMO and NEDD8 hydrolases in our library of 123 RNAi lines targeting 54 genes (designated herein as *DUB^RNAi^* for simplicity) (Table S1). To avoid potential early lethality phenotypes, we regulated RNAi expression spatially and temporally using an *act-Gal4/Gal80^ts^* module and a *FLP/FRT STOP* cassette (FLPout) (Fig. S1A). We expressed the FLPase enzyme under the control of the eye-specific *eyeless* promoter (*ey-FLP*), such that *DUB^RNAi^* expression was limited to the developing eye and was induced by shifting larvae from 18°C to 29°C 120 h after egg laying (AEL) to inhibit *Gal80^ts^* function.

**Fig. 1.**
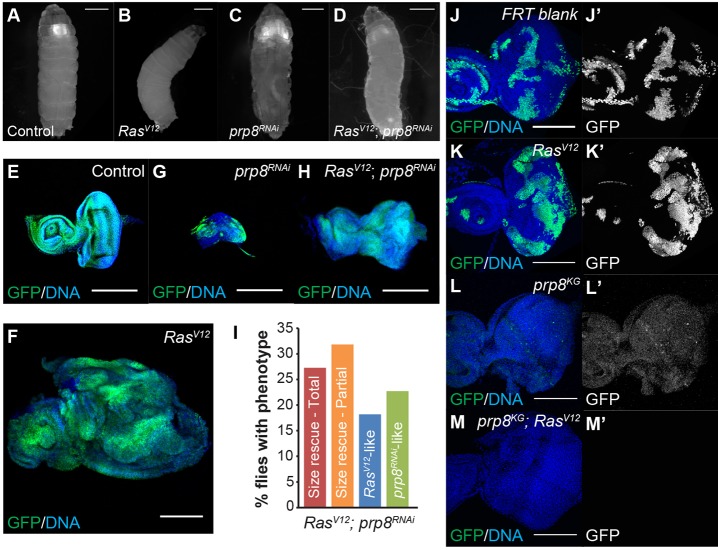
***prp8* knockdown regulates growth during development and tumourigenesis.** (A-D) Images of third instar larvae showing distribution of GFP expression induced in the eye discs and optic lobes of control (A), *Ras^V12^* (B), *prp8^RNAi^* (C) and *Ras^V12^; prp8^RNAi^* (D). (E-H) Confocal micrographs of eye imaginal discs from third instar larvae of the indicated genotypes, stained with anti-GFP (green) and the DNA marker Hoechst (blue). When compared with controls (A and E), *prp8^RNAi^* decreased the GFP-positive area in whole larvae (C) and caused eye disc hypoplasia (G). When combined with *Ras^V12^* expression, *prp8* depletion led to a decrease in the GFP-positive area (D) and partially rescued eye disc morphology (H), when compared with *Ras^V12^* alone (B and F). (I) Quantification of frequency of phenotypes observed with all *prp8^RNAi^* lines used in this study (*n*>60). (J-M) *xy* sections of third instar eye discs containing clones that were induced with the MARCM system. MARCM *FRT42D* blank clones (J), *UAS-Ras^V12^* MARCM clones (K), *prp8^KG03188^* MARCM clones (L) and *prp8^KG03188^*; *UAS-Ras^V12^* MARCM clones (M) are marked by GFP (green). DNA is stained with Hoechst (blue). Scale bars: 1 mm in A-D; 200 µm in E-H; 100 µm in J-M.

We initially assessed the role of DUBs in the normal growth of the developing *Drosophila* eye, and identified three genes which, when depleted, caused eye disc hypoplasia: *prp8* (Fig. 1C,G), *usp10* (Fig. S1B) and *npl4* (Fig. S1C). We selected Prp8 for further study as the hypoplasia phenotype was fully penetrant, and was observed in several RNAi lines that target *prp8*, which were predicted to not have off-target effects. We next tested whether DUBs could influence tumour growth. To this end, we co-expressed the *DUB^RNAi^* library with an oncogenic form of Ras (*Ras^V12^*), thereby mimicking a well-established *Drosophila* tumour model in which expression of *Ras^V12^* causes hyperplasia (Lee et al., 1996; Pagliarini and Xu, 2003) (Fig. S1D,L and Table S2). This *Ras^V12^* model has been used to identify new regulators of growth and metastasis and, for example, previous research has uncovered the fact that combining *Ras^V12^* expression with loss-of-function mutations for polarity genes causes metastasis in larvae (Chabu et al., 2017; Ohsawa et al., 2012; Pagliarini and Xu, 2003). To validate our genetic model, we co-expressed *Ras^V12^* with RNAi lines that target the polarity genes *scribbled* (*scrib^RNAi^*), *lethal giant larvae* (*lgl^RNAi^*) and *bazooka* (*baz^RNAi^*). Consistent with previous reports, a combination of *Ras^V12^* with RNAi against polarity genes resulted in enhanced overgrowth phenotypes in eye discs and, in some cases, in the appearance of distant metastases (compare Fig. S1D with Fig. S1E-G). Therefore, our model mimicked previously used systems to study oncogene-mediated growth and metastasis, and is an appropriate setting to test the role of DUBs in these processes.

Analysis of *Ras^V12^*-expressing eye discs with simultaneous depletion of *prp8* (Fig. 1D,H) revealed a dramatic reduction of eye disc overgrowth and a partial rescue of disc morphology when compared with *Ras^V12^* expression alone (Fig. 1B,F). Indeed, the major effect of *prp8* depletion in *Ras^V12^* tumours was a decrease in size, such that the GFP-positive area in developing eye discs appeared to be similar to that of the controls (compare Fig. 1E with H, quantified in Fig. S4K). When analysed collectively, *prp8^RNAi^* lines led to decreased hyperplastic growth in ∼80% of cases, with some of these eye discs displaying a near rescue of eye disc morphology (∼25% of cases) (Fig. 1I and Fig. S1L). In ∼20% of eye discs we still observed tumours and, in rare cases (8.5%), *prp8* depletion in *Ras^V12^* tumours caused metastases to appear in developing larvae (Fig. S1L). For the majority of our experiments, we used a *prp8^RNAi^* line that resulted in a higher frequency of complete rescue of *Ras^V12^*-induced hyperplasia (*prp8^RNAi 18567GD^*). Importantly, although we detected a significant percentage of tumours in whole larvae when this RNAi line was combined with *Ras^V12^*, upon eye disc dissection in subsequent experiments, the vast majority of *Ras^V12^; prp8^RNAi^* tissues were significantly smaller than *Ras^V12^*-expressing tissues, suggesting that our analysis of intact larvae in fact overestimates the existing tumour growth. We confirmed the effect of *prp8* using the MARCM system to combine loss-of-function of *prp8* (*prp8^KG03188^*) with expression of *Ras^V12^*. Our experiments revealed that, as in the *prp8^RNAi^*, *prp8* loss-of-function clones are smaller than control clones (compare Fig. 1J with 1L). Moreover, combining *Ras^V12^* with *prp8* loss-of-function resulted in a phenotype similar to *prp8* loss alone (compare Fig. 1M with 1L). Together, our results suggest that Prp8 influences *Ras^V12^*-mediated hyperplastic growth and that, to a large extent, depletion of *prp8* impairs *Ras^V12^*-mediated hyperplasia.

### Prp8 controls cell proliferation and cell death

**Fig. 2.**
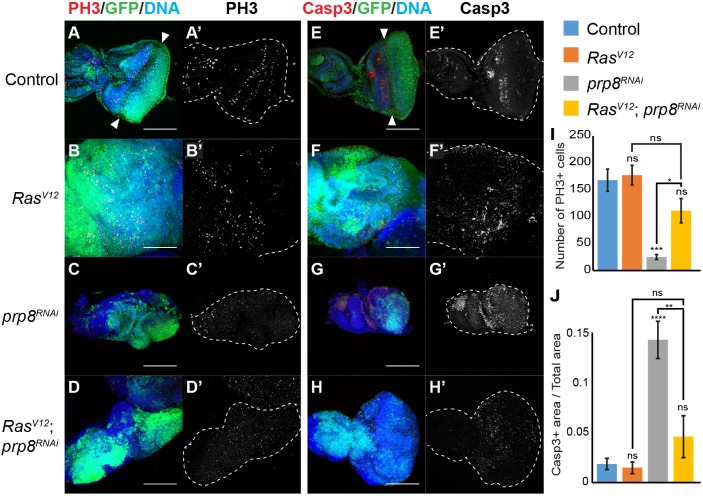
**Prp8 modulates eye disc development by controlling cell proliferation and cell death.** (A-H) Confocal micrographs of eye imaginal discs of the indicated genotypes, labelled with anti-GFP (green), Hoechst (blue) and either anti-phospho Histone H3 (PH3) (A-D, red) or anti-cleaved Caspase 3 (Dcp1) (E-H, red). (I) Quantification of number of PH3-positive cells in eye discs (*n*>5 discs/genotype). (J) Quantification of the ratio between Caspase 3-positive area and total eye disc area (*n*>6 discs/genotype). PH3 was mainly detected adjacent to the morphogenetic furrow (arrowheads) in controls (A and A′). In *Ras^V12^*-expressing discs, the PH3 pattern was mislocalised (B,B′). *prp8* knockdown resulted in fewer PH3-positive cells (C,C′ and I), whereas combining *Ras^V12^* with *prp8^RNAi^* resulted in an intermediate phenotype (D,D′ and I). *prp8^RNAi^* increased cell death (G,G′ and J), which was partially rescued by co-expression with *Ras^V12^* (H,H′ and J). Dashed outlines indicate the outline of the eye disc. Data are mean±s.e.m. *P<0.05; **P<0.01; ***P<0.001; ****P<0.0001 (one-way ANOVA analysis). ns, non-significant. Scale bars: 100 μm.

To elucidate how Prp8 regulates tissue growth, we tested whether the eye disc hypoplasia phenotype obtained with depletion of Prp8 was because of cell proliferation defects. For this, we assessed the levels of phosphohistone-H3 (PH3), a marker of cells that are undergoing mitosis. When compared with controls (Fig. 2A), *prp8^RNAi^* discs displayed reduced cell proliferation (Fig. 2C, quantified in Fig. 2I). In contrast, the number of PH3-positive cells seen in discs that expressed *Ras^V12^* (Fig. 2B) or the *Ras^V12^; prp8^RNAi^* combination (Fig. 2D) was similar to controls (Fig. 2A, quantified in Fig. 2I). We also analysed the G2/M cyclin, Cyclin B (CycB) (Fig. S2A-D) and found that, in both *Ras^V12^* and *prp8^RNAi^* samples, CycB distribution was altered. CycB levels in the presumptive second mitotic wave were reduced in *Ras^V12^*-expressing cells (Fig. S2B), whereas in *prp8^RNAi^* the sharp boundary of CycB expression was lost and its expression was more uniform throughout the disc (Fig. S2C). In both cases, the morphogenetic furrow is absent. Interestingly, depleting *prp8* in *Ras^V12^*-expressing cells leads to a partial rescue of the CycB phenotype and the appearance of a rudimentary morphogenetic furrow (Fig. S2D). We also analysed the number of cells entering S phase by assessing 5-bromo-2′-deoxyuridine (BrdU) incorporation (Fig. S2E) and found that, as expected, *Ras^V12^* increased the number of BrdU-positive cells, which was suppressed when combined with *prp8^RNAi^*. These results suggest that the eye phenotypes that are associated with *prp8* depletion may be due to cell proliferation defects, which is consistent with a previous report that stated *prp8* depletion causes a G2/M arrest (Andersen and Tapon, 2008). Depleting *prp8* from *Ras^V12^*-expressing tissues reduced entry into S phase but not progression through mitosis, as there was no significant difference in the number of PH3-positive cells between *Ras^V12^* and *Ras^V12^; prp8^RNAi^* tissues. Therefore, the effect of Prp8 on cell proliferation appears to be insufficient to explain why the loss of Prp8 blocks *Ras^V12^*-induced hyperplasia.

These observations raise the possibility that Prp8 may affect cell survival. We assessed this using an antibody that recognises cleaved caspases (anti-Dcp1 antibody) and, therefore, reflects the overall level of cell death. Control eye discs have a relatively low level of cell death, which is mostly restricted to the area that juxtapose the morphogenetic furrow (Fig. 2E, quantified in Fig. 2J) (Rusconi et al., 2000). Expression of *Ras^V12^* alone did not significantly alter cell death levels (Fig. 2F,J). In contrast, *prp8^RNAi^* led to a dramatic increase in caspase staining (Fig. 2G,J). Combining *Ras^V12^* with *prp8* depletion resulted in a significant decrease in the levels of active caspase compared with *prp8^RNAi^* alone, which suggests that *Ras^V12^* can rescue the cell autonomous defects that lead to cell death when *prp8* is depleted (Fig. 2H,J). Nevertheless, depletion of *prp8* in *Ras^V12^* tumours resulted in reduced hyperplastic growth (Fig. 2H). Together, these results suggest that loss of Prp8 function leads to defects in eye disc morphology due to a combination of decreased proliferation and increased cell death. However, when oncogenic Ras is present, Prp8 blocks hyperplasia, despite only having a modest effect on cell proliferation and cell death levels relative to *Ras^V12^* alone. To confirm that cell death alone cannot explain the phenotypes associated with loss of Prp8, we used the caspase inhibitor P35. Expression of *P35* in controls did not result in any overt changes in tissue size or number of GFP-positive cells in developing eye discs (Fig. S2F). Consistent with our hypothesis, co-expression of *prp8^RNAi^* and *P35* was insufficient to fully rescue the hypoplasia phenotype that was seen when *prp8* was lost, despite reducing the levels of activated caspases (compare Fig. S2G with Fig. 2G, quantified in Fig. S2H). Therefore, we conclude that although cell death contributes to the *prp8^RNAi^* phenotype, there appear to be other processes that are regulated simultaneously (including cell proliferation) that contribute to regulation of developmental and *Ras^V12^*-induced hyperplastic growth.

### Prp8 regulates cell differentiation in the developing *Drosophila* eye

**Fig. 3.**
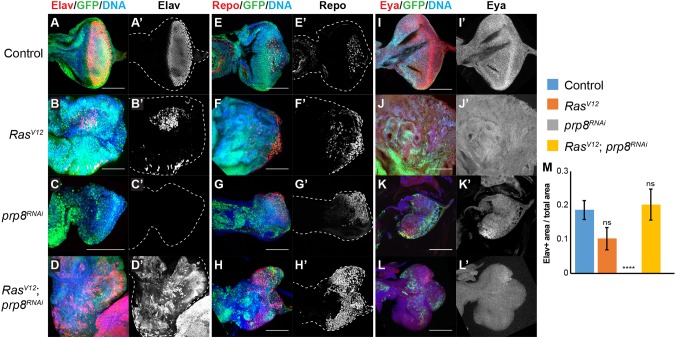
***prp8* depletion induces cell differentiation in hyperplastic tumours.** (A-L) Confocal micrographs of eye imaginal discs of the indicated genotypes, labelled with anti-GFP (green), Hoechst (blue) and either anti-Elav (A-D, red), anti-Repo (E-H, red) or anti-Eya (I-L, red). Both Elav and Repo are located in the posterior region of control eye discs (A,A′ and E,E′). *Ras^V12^* inhibited differentiation to a large extent (B,B′ and F,F′). *prp8^RNAi^* discs lost Elav expression (C,C′), whereas Repo staining remained largely unaffected (G,G′). Co-expression of *Ras^V12^* and *prp8^RNAi^* resulted in an increase in the number of Elav (D,D′) or Repo (H,H′) positive cells, when compared with *Ras^V12^* alone. The pattern of Eya expression in controls (I,I′) is severely disrupted in *Ras^V12^* (J,J′) or *prp8^RNAi^* (K,K′) discs. (M) Quantification of the ratio between Elav-positive area and total eye disc area (*n*>4 discs/genotype). Dashed outlines indicate the outline of the eye disc. Data are mean±s.e.m. ****P<0.0001 (one-way ANOVA analysis). ns, non-significant. Scale bars: 100 μm.

We next assessed whether Prp8 could regulate other processes that influence eye disc development. We first tested cell differentiation, as Prp8 has been associated with differentiation defects (Keightley et al., 2013; Wu et al., 2016) and, in *Drosophila*, eye disc development involves close coupling of cell proliferation, death and differentiation (Cagan, 2009). Moreover, crucial eye disc determinants are thought to be regulated via alternative splicing events (Fic et al., 2007; Roignant and Treisman, 2010), a process for which Prp8 function is crucial (Grainger and Beggs, 2005). To assess whether Prp8 regulates cell differentiation in eye discs, we stained for the photoreceptor differentiation marker Embryonic lethal abnormal vision (Elav, an RNA-binding protein that acts as a neuronal marker), the transcription factor Reversed polarity (Repo, which is restricted to glial cells) and the transcriptional co-activator Eyes absent (Eya, expressed in progenitors before differentiation) (Bonini et al., 1993; Lee and Jones, 2005; Soller and White, 2004). In controls, Elav and Repo were detected primarily in the region posterior to the morphogenetic furrow, which determines the ‘front’ of the cell differentiation wave (Fig. 3A,E). In *prp8^RNAi^* discs, Elav staining was completely lost (Fig. 3C). Repo staining was still detectable in *prp8*-depleted discs, but the localisation and morphology of Repo-positive cells was dramatically changed (Fig. 3G). In contrast, whereas some *Ras^V12^*-expressing cells maintained Elav and Repo expression, the majority were negative for these differentiation markers and, therefore, are presumably undifferentiated (Fig. 3B,F). Interestingly, when *Ras^V12^* was combined with *prp8^RNAi^*, both Elav and Repo were expressed in the overgrown tissue and, when compared with *Ras^V12^* alone, these tissues appeared to have a higher percentage of Elav-positive and Repo-positive cells and resembled the control situation (Fig. 3D,H, quantified in 3M). With regard to Eya, its expression pattern was disrupted in *Ras^V12^*- (Fig. 3J) and *prp8^RNAi^*-expressing tissues (Fig. 3K), compared with controls (Fig. 3I) but, contrary to Elav and Repo, this was not rescued in the *Ras^V12^; prp8^RNAi^* combination (Fig. 3L), indicating that the effect of *prp8* may be limited to specific differentiation markers.

We also assessed whether *prp8* depletion affected signalling downstream of *Ras^V12^*, as it has been previously reported that alterations in spliceosome genes cause dramatic changes in the splicing pattern of MAPK (Ashton-Beaucage et al., 2014). We analysed total and phospho-MAPK levels *in vivo* (Fig. S3A-J) and splicing changes in S2 cells (Fig. S3K and L) and found minor changes, under the conditions tested. Accordingly, expression of MAPK (*rolled*; *rl*) in *prp8^RNAi^*-expressing tissues was unable to rescue *prp8^RNAi^*-mediated hypoplasia, despite the fact that the eye disc morphology was partially rescued (Fig. S3M-P). Therefore, it is unlikely that the effect of *prp8* in *Ras^V12^* hyperplasia is due to an effect on MAPK regulation.

Our data suggest that Prp8 can regulate cell differentiation in developing eye discs and that, in the presence of oncogenic Ras, absence of Prp8 function prevents uncontrolled growth, not by affecting MAPK signalling downstream of Ras but, at least in part, by causing premature or enhanced differentiation of cells. This would render the cells postmitotic, thereby limiting the growth of *Ras^V12^* hyperplastic tissues.

### Prp8 regulates oncogenic tissue morphology in part by affecting cell polarity

**Fig. 4.**
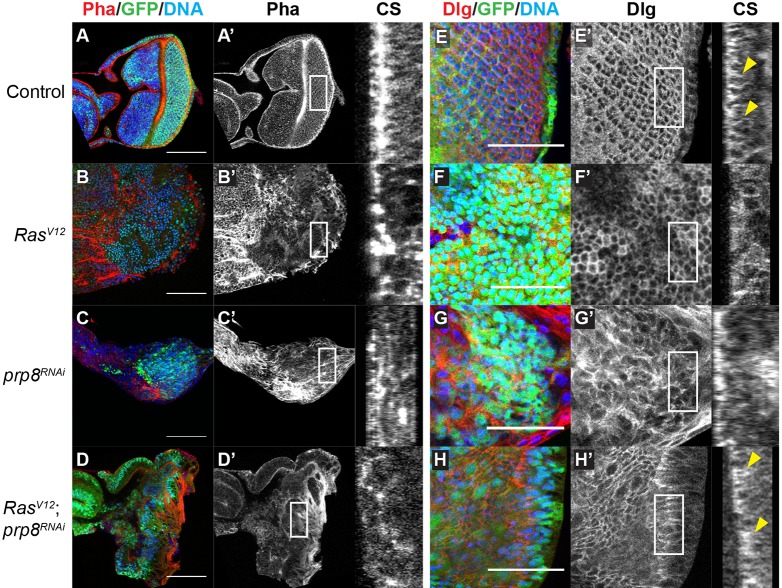
***prp8* regulates cell polarity but not actin localisation in Ras^V12^-induced hyperplasia.** (A-D) Confocal micrographs depicting eye disc morphology for the indicated genotypes. Eye disc morphology was assessed by staining F-actin using rhodamine-conjugated phalloidin (Pha, red). *Ras^V12^* expression caused severe morphology defects and disruption of the F-actin pattern (B,B′). *prp8^RNAi^* induced eye disc hypoplasia and F-actin mislocalisation (C,C′), which persisted when combined with *Ras^V12^* (D,D′). (E-H) Confocal micrographs of eye discs of the indicated genotypes stained for anti-Dlg (red). *Ras^V12^* expression caused spreading of Dlg to the entire cell perimeter (F,F′). Whereas *prp8^RNAi^* alone resulted in Dlg mislocalisation (G,G′), combined expression with *Ras^V12^* rescued the localisation of Dlg (H,H′). CS denotes cross-section images (indicated by boxed areas in A′-H′). Yellow arrowheads denote regions in which the Dlg pattern is similar in controls and *Ras^V12^;* *prp8^RNAi^* tissues. Scale bars: 100 μm in A-D; 40 μm in E-H.

Our data suggest that *prp8^RNAi^* can partially rescue the overall morphology of *Ras^V12^* hyperplastic eye discs (Fig. 1). Therefore, we hypothesised that Prp8 regulates cell processes and the components that are crucial for establishing and/or maintaining tissue morphology, such as polarity and the actin cytoskeleton (Pickup et al., 2002). To address this, we performed immunofluorescence staining for F-actin and the polarity protein Discs large (Dlg) in developing eye discs (Fig. 4). F-actin has a stereotypical organisation, with a prominent accumulation in the morphogenetic furrow and at the periphery of the posterior region of the disc (Fig. 4A). In *Ras^V12^* discs, the loss of overall tissue organisation and structure is reflected in the localisation of F-actin. F-actin is abnormally accumulated in large patches, which are adjacent to regions in which total F-actin appears to be significantly downregulated (Fig. 4B). Surprisingly, *prp8^RNAi^*-associated eye disc hypoplasia was not accompanied by prominent changes in F-actin organisation beside an interruption of the F-actin signal in lateral membranes (Fig. 4C). In *prp8*-depleted eye discs, F-actin still accumulates at the periphery of the posterior region of the disc (Fig. 4C). When *Ras^V12^* and *prp8^RNAi^* were combined (Fig. 4D), we observed that, despite a significant rescue of overall tissue morphology and tissue size, the F-actin pattern was still disorganised and did not resemble either wild-type (Fig. 4A) or *prp8^RNAi^* phenotypes (Fig. 4C). Our results suggest that Prp8-mediated regulation of hyperplasia is mostly independent of a potential minor role of Prp8 in the modulation of the actin cytoskeleton. This hypothesis is consistent with our observation that F-actin structure is still disorganised when *prp8* is depleted from *Ras^V12^*-expressing hyperplastic tissue.

We next examined whether modulation of cell polarity could explain the rescue of tissue morphology that was seen when *prp8^RNAi^* was combined with *Ras^V12^*. Dlg is a basolateral polarity determinant that localises to septate junctions and is visualised in the lateral side of cells (Fig. 4E). Expression of *Ras^V12^* results in Dlg mislocalisation, which appears to spread throughout the cell membrane (Fig. 4F). Loss of *prp8* leads to a dramatic increase in Dlg levels, loss of apico-basal polarity and epithelial organisation (Fig. 4G). When combined with *Ras^V12^*, *prp8^RNAi^* partially rescued Dlg localisation, which is more lateral than in *Ras^V12^* tumours alone, recapitulating the situation seen in the controls (compare Fig. 4H with Fig. 4E and Dlg localisation in cross-section images). Collectively, these results indicate that Prp8 regulates levels and/or localisation of Dlg and this modulation of cell polarity function may be, at least in part, responsible for the rescue of tissue morphology seen during hyperplasia.

### Prp8-mediated regulation of oncogenic growth is not tissue-specific

Next, we determined whether the effect of Prp8 in the regulation of oncogene-induced hyperplasia was a tissue-specific function or whether it was limited to the regulation of *Ras^V12^*-induced hyperplasia. For this, we generated alternative hyperplasia models in the developing eye using activated versions of the EGF receptor (*Egfr^λtop^*) and Notch (*N^ΔECD^*) (Pallavi et al., 2012; Queenan et al., 1997) (Fig. S4). Similar to *Ras^V12^*, expression of EGFR (Fig. S4A) or N (Fig. S4C) led to overgrowth phenotypes. *prp8* depletion significantly reduced the hyperplasia caused by both genes and, in some cases, produced a dramatic rescue of tissue organisation (Fig. S4B,D). Significantly, this suggests that *prp8* regulates hyperplastic growth that is induced by different oncogenes. Moreover, we tested whether the effect of *prp8* on *Ras^V12^*-induced hyperplastic growth was a general role for the spliceosome by depleting the expression of alternative spliceosome components, such as Mfap1 (part of the spliceosome complex B; Fig. S4E,F), Prp38 (part of complex B; Fig. S4G,H) and Bx42 (part of complexes B, C and P; Fig. S4I,J). We found that all the spliceosome components efficiently suppressed *Ras^V12^*-induced hyperplasia in the eye, suggesting that, at least in this tissue, the effect of *prp8* is likely to be mediated by its role in the spliceosome (Fig. S4E-J, quantified in Fig. S4K).

**Fig. 5.**
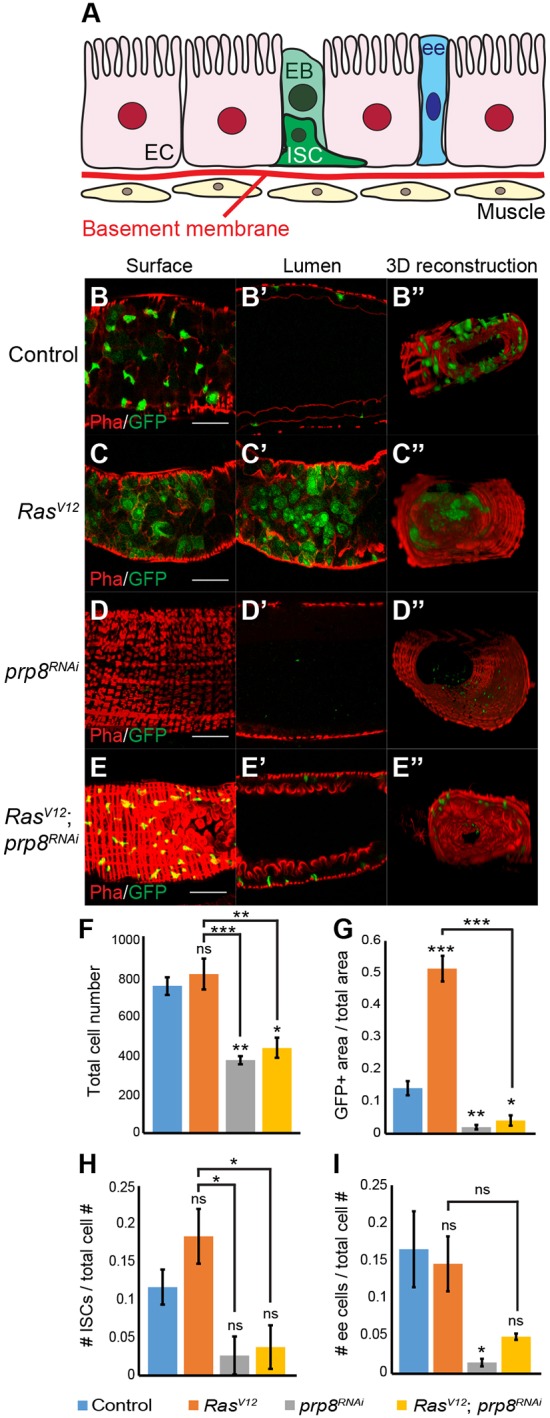
**Prp8 regulates intestinal stem cell dynamics and prevents Ras-induced hyperplasia in the adult gut.** (A) Schematic of adult gut structure, including the different cell types present: intestinal stem cells (ISC), enteroblasts (EB), enteroendocrine cells (EE), enterocytes (EC) and the underlying basement membrane and muscle layer. (B-E) Confocal micrographs of posterior midguts from adult flies of the indicated genotypes, stained for GFP (green) and F-actin (phalloidin, Pha, red). Shown are surface plane images (B-E), lumen sections (B′-E′) and 3D reconstructions (B′′-E′′) of the posterior midgut region. (F) Quantification of total number of cells in the posterior midgut, 7 days after induction (*n*>9 guts/genotype). (G) Quantification of the ratio between the GFP-positive area and total area of the posterior midgut, 7 days after induction (*n*>12 guts/genotype). (H) Quantification of the ratio between the number of Delta-positive (Dl, ISC marker) cells and total number of cells in the posterior midgut, 7 days after induction (*n*>4 guts/genotype). (I) Quantification of the ratio between the number of enteroendocrine cells [Prospero (Pros)-positive] and total number of cells in the posterior midgut, 7 days after induction (*n*>6 guts/genotype). Data are mean±s.e.m. *P<0.05; **P<0.01; ***P<0.001 (one-way ANOVA analysis). ns, non-significant. Scale bars: 50 μm. For B″-E″, the images represent an area of 206.18 μm^2^.

We also assessed whether Prp8 regulated *Ras^V12^*-induced hyperplasia in other tissues. To test this, we selected the adult gut as a model, as expression of *Ras^V12^* in intestinal stem cells (ISC) is known to cause tissue hyperplasia (Ragab et al., 2011; Jiang et al., 2011). The adult gut is maintained by ISCs, which can be identified by the expression of the N ligand Delta (Dl) and small nuclear size. ISCs give rise to enteroblasts (EB) that can differentiate into either enteroendocrine cells (ee) or absorptive enterocytes (EC) (Fig. 5A). Both ISCs and EBs express the transcription factor gene *escargot* (*esg*) and, in our experiments, we combined *esg-Gal4* with a temperature-sensitive version of *Gal80* to control gene expression (Fig. 5A) (Micchelli and Perrimon, 2006; Ohlstein and Spradling, 2006). When compared with controls (Fig. 5B), *esg-Gal4*-mediated expression of *Ras^V12^* in the adult gut led to an increase in the relative area of GFP-positive cells (ISCs and EBs) in the posterior midgut, 7 days after induction (Fig. 5C, quantified in 5G). The total cell number (Fig. 5F) and, in relative terms, the number of GFP-positive cells were similar to controls (Fig. S5I), which suggests that most of the GFP-positive area is because of large GFP-positive cells, which are likely to be ECs in which GFP expression now persists or progenitor cells that become enlarged. Accordingly, the number of small GFP-positive cells (ISCs and/or progenitors) is similar to control (Fig. S5J). *Ras^V12^*-mediated hyperplasia influenced tissue architecture and, in some cases, caused GFP-positive cells to invade the gut lumen (Fig. 5C). In contrast, *prp8* depletion resulted in a significant reduction in GFP-positive area (Fig. 5D,G), accompanied by a significant reduction in the relative number of GFP-positive cells (Fig. S5I). Similar to what we observed in the developing eye, co-expression of *Ras^V12^* and *prp8^RNAi^* resulted in a significant reduction in the levels of *Ras^V12^*-induced hyperplasia (Fig. 5E,G and Fig. S5I), which suggests that Prp8 influences *Ras^V12^*-mediated growth in different tissues.

**Fig. 6.**
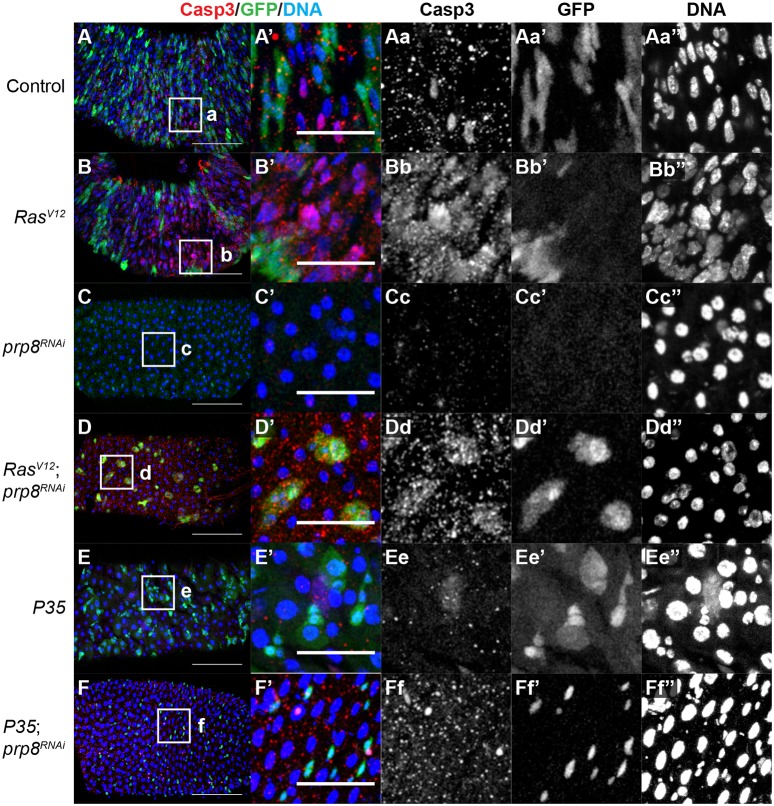
**Blocking caspase activity is not sufficient to restore ISC levels in *prp8^RNAi^* adult guts.** (A-F) Confocal micrographs of posterior midguts from adult flies of the indicated genotypes, stained for cleaved Caspase 3 (Casp3; red in A-F, grey in Aa-Ff), GFP (green in A-F, grey in Aa′-Ff′) and DNA (blue in A-F, grey in Aa′′-Ff′′) 2 days after induction of gene expression. Boxed areas (a-f) indicate regions of interest shown in magnified images (Aa-Ff). Note that P35 reduced Casp3 levels (E) but this did not abrogate the effect of *prp8^RNAi^* on the number of ISCs (F). Scale bars: 100 μm in posterior midgut images A-F; 40 μm in magnified images A′-Ff′′.

Next, we assessed whether *prp8* depletion also affected cell proliferation, differentiation and death in the adult gut. Therefore, we stained adult guts with the ISC marker Dl (Fig. 5H and Fig. S5A-D), the ee marker Prospero (Pros) (Fig. 5I and Fig. S5E-H) and an antibody against active caspases (Fig. 6 and Fig. S6). Expression of *Ras^V12^* did not significantly change the ratio of Dl-positive cells in the gut, which suggests that although *Ras^V12^* promotes the proliferation of ISCs or uncommitted progenitors, these are likely still differentiating appropriately (Fig. 5H and Fig. S5B). In contrast, *prp8^RNAi^*, alone or in combination with *Ras^V12^*, led to a significant decrease in the ratio of Dl-positive cells relative to *Ras^V12^* alone, suggesting that it blocks *Ras^V12^* hyperplasia by blocking ISC and/or progenitor proliferation, promoting their differentiation or inducing apoptosis (Fig. 5H, Fig. S5C,D). With regard to ee cells, *Ras^V12^* expression had no effect on the proportion of ee cells in the adult gut, which suggests that the increased proliferation of ISCs and/or progenitors does not result in increased levels of differentiation to the ee lineage (Fig. 5I and Fig. S5F). In contrast, the proportion of ee cells was markedly reduced in *prp8^RNAi^*-expressing guts (Fig. 5I, Fig. S5G and H). As the proportion of ee cells decreased when Prp8 was absent, these results suggest that it is unlikely that Prp8 loss blocks *Ras^V12^* tumours by promoting the differentiation of ee cells.

Finally, we analysed the levels of activated caspases in the posterior midgut at different times after gene induction to determine whether modulation of caspase-dependent cell death could explain the phenotypes that are seen with *prp8^RNAi^* alone or in combination with *Ras^V12^*. In controls (Fig. S6A), the levels of apoptosis 4 days after induction were low and the majority of caspase-positive cells were GFP-negative (therefore not *esg-Gal4* positive), which is in agreement with the fact that most of the remodelling in the gut happens at the level of ECs and ee cells (Amcheslavsky et al., 2009; Jiang et al., 2009). In guts that expressed *Ras^V12^*, there was an increase in the number of caspase-positive cells, which were found both in areas that contained GFP-positive cells and in adjacent GFP-negative areas (Fig. S6B). This suggests that *Ras^V12^* increases cell proliferation but also cell death, both in ISCs/progenitors and GFP-negative cells. This would explain why the total number of cells in the posterior midgut was similar between controls and *Ras^V12^* (Fig. 5F). When we depleted *prp8* using the *esg-Gal4* driver, we observed very low levels of caspase staining 4 days after induction (Fig. S6C). This was also the case when *prp8* was depleted in combination with *Ras^V12^* expression (Fig. S6D). At later stages (7 days after transgene induction), the number of caspase-positive cells was low in all genotypes (data not shown). Therefore, we assessed an earlier time point, 2 days after induction, which yielded similar results (Fig. 6A-D). However, in this situation we observed caspase activity in GFP-positive cells of *Ras^V12^; prp8^RNAi^* animals, which indicated a potential loss of progenitor cells (Fig. 6D). In addition, we tested whether blocking apoptosis using P35 could rescue the reduction in the number of GFP-positive cells that was seen when *prp8* was depleted. Expression of *P35* alone reduced active caspase levels but had no overt effect on the adult gut 2 days after induction (Fig. 6E). Flies that expressed *prp8^RNAi^* and *P35* displayed phenotypes that were similar to *prp8^RNAi^* alone (compare Fig. 6C with F), which indicates that blocking cell death is insufficient to rescue the *prp8^RNAi^* phenotype. These results suggest that either Prp8 does not affect cell death in these conditions or that cell death occurs at an earlier time point and/or is caspase-independent. Depleting *prp8* in the context of *Ras^V12^* hyperplasia was associated with cell death, but it was not overtly different from the caspase activation that was seen in *Ras^V12^*-expressing tissues. Together, our data suggest that Prp8 reduces *Ras^V12^*-mediated hyperplastic growth in different tissues, potentially using context-dependent tissue-specific mechanisms (i.e. modulation of proliferation and differentiation in the eye versus modulation of proliferation in the gut).

### Prp8 antagonises RET-induced cell invasion

**Fig. 7.**
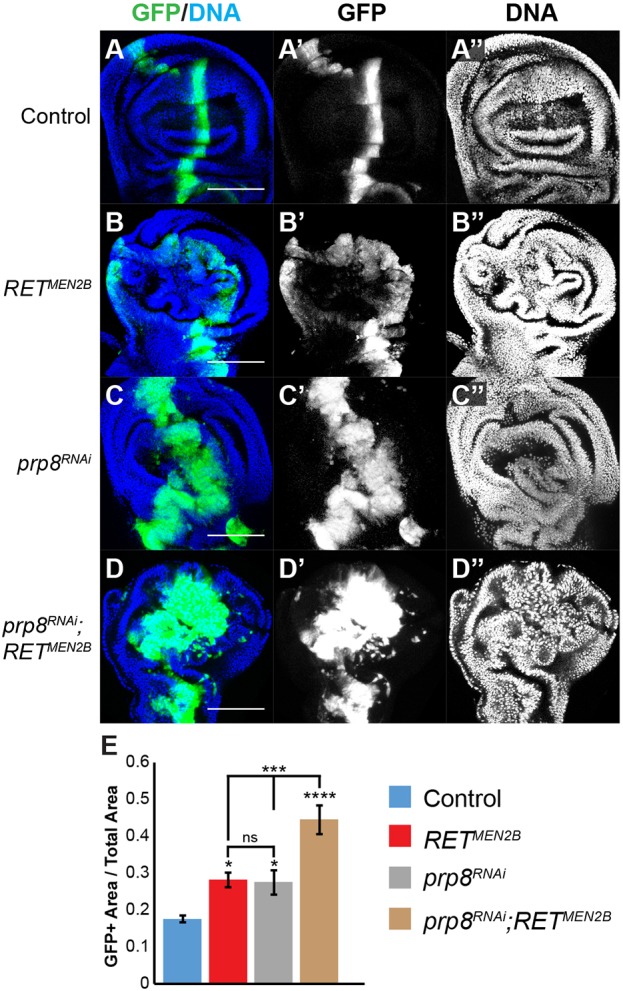
**Loss of *prp8* enhances the proliferation and invasion phenotype of oncogenic RET.** (A-D) Confocal micrographs of wing imaginal discs from third instar larvae of the indicated genotypes, stained for GFP (green) and DNA (blue). GFP expression marks the anterior-posterior boundary and the *ptc-Gal4*-expressing domain. *RET^MEN2B^* expression (B) and *prp8* depletion (*prp8^RNAi^*; C) caused an increase in the GFP-positive area, when compared with controls (A). Combining *RET^MEN2B^* and *prp8^RNAi^* enhanced the *RET^MEN2B^* phenotype and the appearance of invasive cells outside the anterior-posterior boundary (D). (E) Quantification of the ratio between the GFP-positive area and total area of the wing disc in the indicated genotypes (*n*>7 discs/genotype). Data are mean±s.e.m. *P<0.05; ***P<0.001; ****P<0.0001 (one-way ANOVA analysis). ns, non-significant. Scale bars: 100 µm.

Next, we tested whether Prp8 regulates oncogene-induced hyperplastic growth and invasion in the developing wing disc. For this, we combined *prp8^RNAi^* with expression of an oncogenic version of the receptor tyrosine kinase RET (*RET^MEN2B^*; M955T point mutation) in the anterior-posterior boundary of the developing wing disc using *patched-Gal4* (*ptc-Gal4*), as this system has previously been used to study invasion and metastasis and the receptor tyrosine kinase RET (Das et al., 2013; Read et al., 2005; Rudrapatna et al., 2012). Activating mutations in RET lead to the cancer syndrome multiple endocrine neoplasia type 2, which is associated with the occurrence of multiple tumours, including the highly metastatic medullary thyroid carcinoma. RET was used in this instance as *ptc-Gal4*-mediated expression of *Ras^V12^* led to early lethality, even when crosses were performed at 18°C (data not shown). As previously shown, when compared with controls (Fig. 7A), expression of oncogenic RET increased the *ptc-Gal4*-expressing area (Fig. 7B, quantified in Fig. 7E) (Das et al., 2013). In contrast, *prp8* depletion resulted in disruption of the wing morphology and the appearance of GFP-positive cells outside of the *ptc* domain, indicative of potential cell invasion, which is in sharp contrast to what we observed in the eye disc and adult gut in the presence of oncogenic Ras (Fig. 7C,E). This phenotype was significantly enhanced when *prp8* depletion was combined with RET expression, with a clear increase in the number of GFP-positive cells outside of the *ptc-Gal4* domain (Fig. 7D,E). These results suggest that, in this tissue, *prp8* may act as a tumour suppressor gene. Alternatively, the distinct outcome that is seen when removing *prp8* function in both settings could be due to the activation of different signalling modules by *Ras^V12^* or oncogenic RET, that is, the function of Prp8 would be context-dependent. We also assessed the levels of caspase activation in this setting and found that both control (Fig. S7A) and RET-expressing wing discs exhibited low levels of caspase activation (Fig. S7B). In agreement with our eye disc data, depletion of *prp8* led to moderate caspase activation in the *ptc*-expressing domain, which is consistent with previous reports (Fig. S7C) (Claudius et al., 2014). Surprisingly, when *prp8^RNAi^* was combined with RET expression, the levels of caspase activation were dramatically increased, particularly in areas that were adjacent to wild-type cells (Fig. S7D). This suggests that the increased invasion of *prp8^RNAi^*; *RET^MEN2B^* cells may be driven by an increase in caspase activity. This hypothesis is in line with previous studies that implicate caspases in cell invasion via the activation of JNK signalling and subsequent activation of matrix metalloproteases that remodel the extracellular matrix and destroy the basement membrane, allowing cells to invade (Rudrapatna et al., 2013). We also assessed whether the effect of Prp8 in RET-induced hyperplasia could be a general feature of affecting spliceosome function. We depleted *mfap1* (Fig. S7E,F), *prp38* (Fig. S7G,H) or *bx42* (Fig. S7I and J) in the *ptc-Gal4* domain and found that, in marked contrast to *prp8* depletion, removing the function of these spliceosome components significantly suppressed the *RET^MEN2B^* phenotype (compare Fig. S7F,H,J with Fig. S7B). Collectively, our results identify Prp8 as a crucial regulator of hyperplastic growth. The precise function of Prp8 in tumours still requires further studies but appears to depend on the driving oncogene and may involve tissue-specific mechanisms, which may be dependent or independent of the function of Prp8 in the spliceosome.

## DISCUSSION

In this report, using an *in vivo* RNAi screening approach, we identify Prp8 as a crucial regulator of oncogene-induced hyperplastic growth in *Drosophila*. Depletion of *prp8* in the developing eye caused significant hypoplasia, suggesting that Prp8 is required for eye disc development. Depleting *prp8* in *Ras^V12^*-expressing tissues suppressed the *Ras^V12^* overgrowth phenotype and, in some cases, resulted in a rescue of the global tissue structure. Despite the fact that *prp8^RNAi^* tissues display increased apoptosis and reduced proliferation, this alone is unlikely to account for all of the phenotypes that were observed when *prp8* was depleted alone or in combination with *Ras^V12^*. Indeed, simply blocking apoptosis in *prp8*-depleted tissues was insufficient to rescue the eye disc hypoplasia phenotype. This suggests that Prp8 has a complex pleiotropic effect during development and in the context of deregulated growth, in line with its role as a crucial spliceosome component (Grainger and Beggs, 2005; Shi, 2017). Accordingly, in tissues that express both *Ras^V12^* and *prp8^RNAi^*, hyperplasia was decreased, despite the fact that apoptosis levels were significantly reduced compared with *prp8^RNAi^* alone. Moreover, the levels of proliferation were not significantly affected. This raises an important question: how does *prp8* depletion suppress hyperplasia in the absence of changes in proliferation and caspase-dependent cell death? One possible explanation is the apparent increase in cell differentiation that is seen in *Ras^V12^; prp8^RNAi^* tissues, which would preclude further proliferation, as cells would enter a postmitotic state (Silies et al., 2010). This is a potentially conserved strategy, as there are examples of differentiation acting as a tumour-suppressive mechanism in mammals, most notably in relation to p53 function and Notch signalling in skin cancer (Bieging et al., 2014; Guinea-Viniegra et al., 2012; Restivo et al., 2011). In several *Drosophila* tissues, *Ras^V12^* expression blocks differentiation, at least in part, by co-opting JNK activity (Brumby et al., 2011; Pastor-Pareja and Xu, 2013; Zeng et al., 2010). Loss of *prp8* function in this context may affect the ability of Ras to block differentiation programs. Indeed, depletion of *prp8* alone led to changes in cell differentiation in the eye disc and a specific loss of neuronal markers. Interestingly, a zygotic Prp8 mutant in zebrafish displayed extensive neuronal cell loss, as well as defects in the differentiation of myeloid cells (Keightley et al., 2013). This observation, combined with the suggestion that genes that are essential for eye disc development undergo alternative splicing, suggests that Prp8 has a conserved function in the regulation of developing neurons and is involved in cell fate decisions (Fic et al., 2007; Keightley et al., 2013; Roignant and Treisman, 2010). Alternatively, depletion of *prp8* in *Ras^V12^*-expressing tissues could influence their ability to outcompete wild-type cells or cause a cell cycle delay that promotes cell differentiation, as it has been previously shown that *prp8* regulates G2/M transition (Andersen and Tapon, 2008).

Interestingly, we also observed a role for *prp8* in the regulation of *Ras^V12^*-induced hyperplasia in the adult gut. Depletion of *prp8* in *Ras^V12^*-expressing tissues significantly reduced hyperplasia and the cells no longer invade the gut lumen. Despite this, we failed to detect increased levels of apoptosis in *Ras^V12^; prp8^RNAi^* cells compared with cells expressing *Ras^V12^* alone, which is consistent with our observations in the eye disc. However, we did not detect cell differentiation changes in the adult gut, at least at the level of the ee population. Our results do not rule out an effect of *prp8* in the regulation of EC differentiation, which could potentially be masked by the fact that the majority of the cell renewal in the gut involves ECs (Guo et al., 2016). However, we would favour the hypothesis that Prp8 affects mostly proliferation or stem cell dynamics, rather than directly affecting differentiation, as the number of GFP-positive cells and ISCs was reduced when *prp8* was depleted. Our data indicate that the precise mechanism by which *prp8* regulates oncogene-induced hyperplasia may differ according to the affected tissue. Consistent with this, despite presumably regulating splicing ubiquitously, mutations in the human orthologue of *prp8*, *PRPF8*, are generally only associated with diseases in specific organs, such as retinitis pigmentosa which affects the eye (Grainger and Beggs, 2005).

Our results also raise issues regarding the role of the spliceosome in the regulation of tumour growth. We found that depletion of spliceosome components resulted in suppression of *Ras^V12^*-induced hyperplasia. This would suggest that *prp8* regulates *Ras^V12^*-mediated hyperplasia through its function within the spliceosome. This would be in agreement with the observation that mutation in other spliceosome components besides *PRPF8* can cause retinitis pigmentosa in humans (Krausova and Stanek, 2017). However, in the context of oncogenic RET, we found that *prp8* depletion enhances the RET phenotype, whereas depleting other spliceosome components suppresses it. This would suggest that Prp8 regulates *RET^MEN2B^*-mediated hyperplasia and invasion in a spliceosome-independent manner, which requires further investigation. Notably, it has been previously shown that other spliceosome components have pleiotropic roles and additional non-splicing-related functions, as exemplified by Prp19 (Chanarat and Strasser, 2013). It will also be important to determine the molecular requirements for the function of *prp8* in the regulation of hyperplasia, that is, which are the protein domains involved. Prp8 is considered to be an inactive DUB because of specific amino acid alterations in the MPN/JAB domain that serves as the catalytic domain in JAMM family DUBs (Clague et al., 2013; Grainger and Beggs, 2005; Komander et al., 2009; Pena et al., 2007). However, as the Prp8 MPN/JAB domain is essential for its function and can bind ubiquitin, it is possible that the role of Prp8 requires this domain and that it may involve the interaction of Prp8 with ubiquitylated proteins (Bellare et al., 2006). Detailed *in vivo* structure-function analysis will be required to fully elucidate this point but, interestingly, previous studies have suggested that ubiquitylation is important for the modulation of spliceosome protein-protein interactions (Das et al., 2017; Song et al., 2010). As for the downstream mechanisms involved, our data do not support a major role for modulation of the MAPK signalling cascade. Despite the fact that previous studies have shown that alterations in spliceosome genes cause dramatic changes in the splicing pattern of MAPK and affect Ras downstream signalling, we failed to uncover a major effect of Prp8 in this process and, accordingly, expressing MAPK in *prp8^RNAi^* tissues was insufficient to rescue their hypoplasia (Ashton-Beaucage et al., 2014).

Prp8 appears to be required for Ras-driven hyperplasia and this role appears to be conserved with other oncogenes, such as activated *Egfr* and *N*. Therefore, our data extends recent observations in the context of human cancers, in which the spliceosome has been identified as a potential therapeutic target in Myc-driven tumours (Hsu et al., 2015). Our results suggest that Prp8 could be a specific target in tumours driven not only by Ras, Notch and EGFR but, potentially, by other receptor tyrosine kinases that signal through Ras and downstream pathways. However, as *prp8* depletion enhanced the phenotype of *RET^MEN2B^* in the developing wing, it will be important to define the mechanisms that control the function of Prp8 (and of the spliceosome) in the regulation of hyperplasia and tumour growth. In conclusion, clearly more work is needed to determine in which conditions inhibiting the spliceosome will be beneficial for cancer treatment.

## MATERIALS AND METHODS

### Fly strains and genetic crosses

MARCM experiments were performed using the *y, w, hsFLP, UAS-GFP-nls; tub-Gal4, FRT42D tub-Gal80* MARCM maker stock. Below are the respective genotypes for the MARCM experiment: *y, w, hsFLP, UAS-GFP-nls; tub-Gal4, FRT42D tub-Gal80/FRT42D blank* (Fig. 1J); *y, w, hsFLP, UAS-GFP-nls; tub-Gal4, FRT42D tub-Gal80/FRT42D blank; +/UAS-Ras^V12^* (Fig. 1K); *y, w, hsFLP, UAS-GFP-nls; tub-Gal4, FRT42D tub-Gal80/FRT42D prp8^KG03188^* (Fig. 1L); and *y, w, hsFLP, UAS-GFP-nls; tub-Gal4, FRT42D tub-Gal80/FRT42D prp8^KG03188^; +/UAS-Ras^V12^* (Fig. 1M).

For additional details of fly strains used, see supplementary Materials and Methods.

### Immunostaining

Third instar larval imaginal discs and adult guts were dissected in PBS and fixed for 20-30 min at room temperature (RT) in PBS containing 4% formaldehyde. After washing with 0.1% Triton X-100 (TX)/PBS, tissues were permeabilised with 0.1% or 0.3% TX/PBS for 30 min and, following additional washing steps with 0.1% TX/PBS (five times for 5 min), blocked for 30 min in blocking buffer [10% normal goat serum (NGS), 0.1% TX/PBS] and incubated overnight at 4°C with primary antibody diluted in blocking buffer. After washing with 0.1% TX/PBS and a blocking step, tissues were incubated for 1-4 h at RT with secondary antibodies. Samples were mounted in Vectashield (Vector Laboratories) after additional washing steps. For F-actin and DNA staining, tissues were incubated with TRITC-conjugated phalloidin (1:500; Sigma-Aldrich) or Hoechst 33342 (1:1000; Thermo Fisher Scientific), respectively, for 15 min during one of the final washing steps.

### Antibodies

The following antibodies were used: rabbit anti-GFP (A-11122; Thermo Fisher Scientific; 1:1000), mouse anti-GFP (A-11120; Thermo Fisher Scientific; 1:1000) or chicken anti-GFP (ab13970; Abcam; 1:1000); rabbit anti-cleaved Caspase 3 (DCP-1) (9578; Cell Signaling Technology, 1:250); rabbit anti-phospho Histone H3 Ser10 (06-570; Merck; 1:1000); mouse anti-Dlg [4F3; Developmental Studies Hybridoma Bank (DSHB); 1:250]; rat anti-Elav (7E8A10; DSHB; 1:10); mouse anti-Repo (8D12; DSHB; 1:10); mouse anti-Delta (C594.9B; DSHB; 1:100); mouse anti-Prospero (MR1A; DSHB; 1:20); mouse anti-BrdU (G3G4; DSHB; 1:10); mouse anti-Cyclin B (F2F4; DSHB; 1:5); mouse anti-Eya (eya10H6; DSHB; 1:100); rabbit anti-MAPK (M5670; Sigma-Aldrich; 1:500) rabbit anti-phospho-MAPK (p44/42 MAPK ERK1/2–137F5; Cell Signaling Technology; 1:500). Secondary antibodies used were coupled to FITC (1:1000), Alexa Fluor 488, Alexa Fluor 568, Alexa Fluor 633, Alexa Fluor 647 (1:2000), Cy3 or Cy5 (1:200 or 1:500, depending on the primary antibody used) (Molecular Probes).

### BrdU analysis

BrdU analysis was performed as previously reported (Chioda et al., 2010). Briefly, wandering third instar larvae were collected and eye imaginal discs were dissected in PBS. Discs were incubated in 1× PBS containing 20 μM BrdU (Sigma-Aldrich) for 30 min. Discs were fixed in PBS with 4% formaldehyde for 30 min at RT. DNA was denatured with 3 M HCl for 30 min. Samples were washed 3× with PBS containing 0.3% TX, followed by incubation in blocking buffer (10% NGS, 0.1% TX/PBS) for 1 h. Samples were incubated with mouse anti-BrdU antibody (G3G4; DSHB; 1:10) overnight at 4°C. Subsequent steps were performed as described in the immunostaining section.

### Analysis of cell numbers in adult gut

Nuclei were segmented in 3D using a user-defined fluorescence threshold and watershed to separate nuclei in contact. Segmented nuclei were then classified into big or small cells based on their volume (the threshold was ∼90 μm^3^ but it was optimised for each image) and within each category they were classified as part of a clone or not based on their mean green intensity value, which was also optimised for each image. To avoid changes in fluorescence owing to the depth of the tissue, only nuclei in the half of the gut closest to the coverslip were considered. The code was implemented in Matlab and is available at github.com/juliafs93/CellCounter.

### *Drosophila* cell culture and expression constructs

*Drosophila* S2 cells were grown in *Drosophila* Schneider's medium (Thermo Fisher Scientific) supplemented with 10% (v/v) foetal bovine serum, 50 μg/ml penicillin and 50 μg/ml streptomycin. Expression plasmids were transfected using Effectene transfection reagent (Qiagen) according to the manufacturer's instructions. Expression plasmids were generated using Gateway technology (Thermo Fisher Scientific). All vectors were verified by sequencing. S2 cells and the Ras^V12^ cDNA were a kind gift from Nic Tapon (The Francis Crick Institute, London, UK).

### dsRNA production and treatment

dsRNAs were synthesised using the Megascript T7 kit (Thermo Fisher Scientific) according to the manufacturer's instructions. DNA templates for dsRNA synthesis were PCR amplified from genomic DNA or from plasmids that encoded the respective genes using primers that contained the 5′ T7 RNA polymerase-binding site sequence. dsRNA primers were designed using the DKFZ RNAi design tool (www.dkfz.de/signaling/e-rnai3). The following primers were used: *lacZ* (forward, TTGCCGGGAAGCTAGAGTAA; reverse, GCCTTCCTGTTTTTGCTCAC) and *prp8* (forward, CGAGTCTGGCTGTTCTTTATGC; reverse, ATGTACGGACCGTCCTTTAAGTAG). After seeding, S2 cells were incubated with 15-20 μg dsRNA for 1 h in serum-free medium, before complete medium was added. Cells were lysed and processed for further analysis 72 h after dsRNA treatment.

### RNA isolation and RT-PCR analysis

Total RNAi was extracted from S2 cells using the QIAshredder and RNeasy kits (Qiagen) according to the manufacturer's protocols. RNA purity and concentration were assessed using a Nanodrop One UV-Vis spectrophotometer (Thermo Fisher Scientific). cDNA was synthesised using the QuantiTect Reverse Transcription kit (Qiagen) following the manufacturer's instructions. RT-PCR analysis was performed using 1 μl of cDNA per PCR reaction and the following primers: MAPK full-length (forward, CGCCGTCGATTTTGATAAATCATATTTACGC; reverse, AGGCGCATTGTCTGGTTGTCGT) (Ashton-Beaucage et al., 2014). RT-PCR products were run in 2% UltraPure agarose (Thermo Fisher Scientific) gels and imaged in an Amersham Imager 600 (GE Healthcare).

### Image acquisition and analysis

For *in vivo* RNAi screen studies, whole larva images were acquired using a Zeiss SteREO Lumar V12 stereomicroscope. Confocal images were acquired at ×20 or ×40 magnification using a Zeiss LSM710 confocal microscope or an Olympus FV1000 confocal microscope equipped with 20×/0.85 oil and 40×/1.35 oil iris objectives. All images were taken as *z*-stacks of 1 µm sections in eye and wing imaginal discs and in the posterior midgut region immediately anterior to the hindgut (R4-R5 region). For cross-sections and 3D reconstructions, images were acquired as *z*-stacks of optimal sections. Image processing, analysis and 3D reconstruction were performed with ImageJ and Imaris XT8.0.

### Quantification and statistical analyses

3D reconstruction images were quantified using Imaris 8.4 and ImageJ and quantifications were performed throughout the volume of the reconstruction. GFP area was calculated in 3D volume using Imaris 8.4 or ImageJ. Quantification of cell numbers, Delta-positive and Prospero-positive cells was performed manually in ImageJ. Statistical analyses were performed in Microsoft Excel or GraphPad Prism. Significance (*P*) values were determined using one-way ANOVA analysis (with Tukey's post test for multiple comparisons or Kruskal–Wallis and Dunn post tests for non-parametric *t*-tests). Unless otherwise stated, data is represented as mean±s.e.m.

## Supplementary Material

Supplementary information
