# Correction: Prp8 regulates oncogene-induced hyperplastic growth in *Drosophila*

**DOI:** 10.1242/dev.205971

**Published:** 2026-08-14

**Authors:** Cecilia H. Fernández-Espartero, Alberto Rizzo, Alexander D. Fulford, Julia Falo-Sanjuan, Damien Goutte-Gattat, Paulo S. Ribeiro

There was an error in *Development* (2018) **145**, dev162156 (doi:10.1242/dev.162156).

The authors contacted the journal to inform us that panels B and C of Fig. 1 had been inadvertently switched during the proof correction process. We have been able to verify this through examination of files provided for Fig. 1 at the time of article acceptance. The corrected Fig. 1 is shown below.

**Fig. 1.**
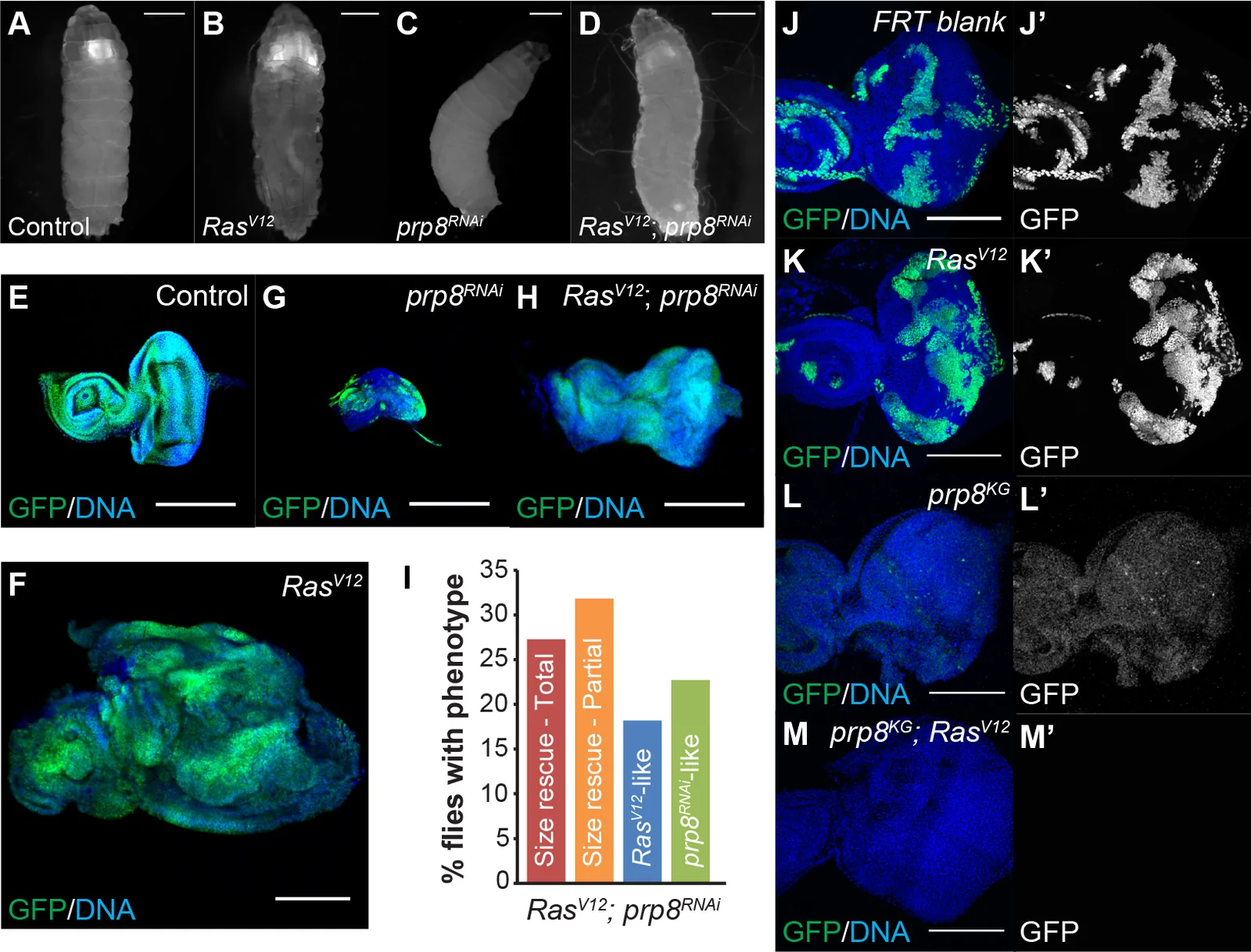
**(corrected). *prp8* knockdown regulates growth during development and tumourigenesis.** (A-D) Images of third instar larvae showing distribution of GFP expression induced in the eye discs and optic lobes of control (A), *RasV^12^* (B), *prp8^RNAi^* (C) and *RasV^12^*; *prp8^RNAi^* (D). (E-H) Confocal micrographs of eye imaginal discs from third instar larvae of the indicated genotypes, stained with anti-GFP (green) and the DNA marker Hoechst (blue). When compared with controls (A and E), *prp8^RNAi^* decreased the GFP-positive area in whole larvae (C) and caused eye disc hypoplasia (G). When combined with *RasV^12^* expression, *prp8* depletion led to a decrease in the GFP-positive area (D) and partially rescued eye disc morphology (H), when compared with *RasV^12^* alone (B and F). (I) Quantification of frequency of phenotypes observed with all *prp8^RNAi^* lines used in this study (*n*>60). (J-M) *xy* sections of third instar eye discs containing clones that were induced with the MARCM system. MARCM *FRT42D* blank clones (J), UAS-*RasV^12^* MARCM clones (K), *prp8^KG03188^* MARCM clones (L) and *prp8^KG03188^*; UAS*RasV^12^* MARCM clones (M) are marked by GFP (green). DNA is stained with Hoechst (blue). Scale bars: 1 mm in A-D; 200 µm in E-H; 100 µm in J-M.

The authors apologise for this error, which does not impact the results and conclusions of the paper.

