# Supplementary material for: Prp8 regulates oncogene-induced hyperplastic growth in Drosophila
Source: Development. 2018 Nov 12;145(22):dev162156. doi: 10.1242/dev.162156 (PMC6262796; doi:10.1242/dev.162156)
Supplement: Supplementary information [file develop-145-162156-s1.pdf]

# Supplementary Materials and Methods

## *Fly stocks*

The *UAS-DUB*$^{RNAi}$ library (Supplementary Table 1) was compiled in collaboration with Pascal Meier (Institute of Cancer Research, UK). *UAS-Ras*$^{V12}$ **(Lee et al., 1996)**, *UAS-EGFR*$^{\lambda Top4.1}$ **(Queenan et al., 1997)**, *UAS-DIAP1*, *UAS-p35* and the Ras$^{V12}$ cDNA were kind gifts from Nic Tapon. *UAS-RET*$^{MEN2B}$ **(Read et al., 2005)** was a kind gift from Ross Cagan. *UAS-N*$^{\Delta ECD}$ was a kind gift from Sarah Bray. The FRT42D MARCM maker stock was a kind gift from Barry Thompson. The *prp8*$^{KG03188}$ allele was obtained from the Kyoto Stock Center (DGRC). Other stocks were obtained from the Bloomington Stock Centre. More information regarding *Drosophila* genes and stocks is available on FlyBase (http://flybase.org).

## *Fly genotypes*

Fig. 1A, 1E, 2A, 2E, 3A, 3E, 3I, 4A, 4E, S2A, S3A, S3E, S3M: *eyFLP; tub-Gal80*$^{ts}$*; act<CD2<Gal4, UAS-GFP*

Fig. 1B, 1F, 2B, 2F, 3B, 3F, 3J, 4B, 4F, S1D, S2B, S3B, S3F: *eyFLP/+; tub-Gal80*$^{ts}$*/UAS-Ras*$^{V12}$*; act<CD2<Gal4, UAS-GFP/TM6B*

Fig. 1C, 1G, 2C, 2G, 3C, 3G, 3K, 4C, 4G, S2C, S3C, S3G, S3N: *eyFLP/+; tub-Gal80*$^{ts}$*/+; act<CD2<Gal4, UAS-GFP/ UAS-prp8*$^{RNAi}$ *(18567GD)*

Fig. 1D, 1H, 2D, 2H, 3D, 3H, 3L, 4D, 4H, S2D, S3D, S3H: *eyFLP/+; tub-Gal80*$^{ts}$*/UAS-Ras*$^{V12}$*; act4<CD2<Gal4, UAS-GFP/UAS-prp8*$^{RNAi}$ *(18567GD)*

Fig. 1J: *y, w, hsFLP, UAS-GFP-nls; tub-Gal4, FRT42D tub-Gal80/FRT42D blank*

Fig. 1K: *y, w, hsFLP, UAS-GFP-nls; tub-Gal4, FRT42D tub-Gal80/FRT42D blank; +/UAS-Ras*$^{V12}$

Fig. 1L: *y, w, hsFLP, UAS-GFP-nls; tub-Gal4, FRT42D tub-Gal80/FRT42D prp8*$^{KG03188}$

Fig. 1M: *y, w, hsFLP, UAS-GFP-nls; tub-Gal4, FRT42D tub-Gal80/FRT42D prp8*$^{KG03188}$*; +/UAS-Ras*$^{V12}$

Fig. 5B, 6A, S5A, S5E, S6A: *yw; esg-Gal4; tub-Gal80*$^{ts}$*, UAS-GFP*

Fig. 5C, 6B, S5B, S5F, S6B: *yw; esg-Gal4/UAS-Ras*$^{V12}$*; tub-Gal80*$^{ts}$*, UAS-GFP/+*

Fig. 5D, 6C, S5C, S5G, S6C: *yw; esg-Gal4/+; tub-Gal80*$^{ts}$*, UAS-GFP/ UAS-prp8*$^{RNAi}$ *(18567GD)*

Fig. 5E, 6D, S5D, S5H, S6D: *yw; esg-Gal4/ UAS-Ras*$^{V12}$*; tubGal80*$^{ts}$*, UAS-GFP/ UAS-prp8*$^{RNAi}$ *(18567GD)*

Fig. 6E: *yw; esg-Gal4/UAS-P35; tub-Gal80*$^{ts}$*, UAS-GFP/+*

Fig. 6F: *yw; esg-Gal4/UAS-P35; tub-Gal80*$^{ts}$*, UAS-GFP/ UAS-prp8*$^{RNAi}$ *(18567GD)*

Fig. 7A, S7A: *w; ptc-Gal4, UAS-CD8-GFP/+*

Fig. 7B, S7B: *w; ptc-Gal4, UAS-CD8-GFP/+; UAS-RET*$^{MEN2B\ (M955T)}$*/+*

Fig. 7C, S7C: *w; ptc-Gal4, UAS-CD8-GFP/UAS-prp8*$^{RNAi}$ *(18565GD)*

Fig. 7D, S7D: *w; ptc-Gal4, UAS-CD8-GFP/ UAS-prp8*$^{RNAi}$ *(18565GD); UAS-RET*$^{MEN2B\ (M955T)}$*/+*

Fig. S1B: *eyFLP/+; tub-Gal80*$^{ts}$*/UAS-Usp10*$^{RNAi}$ *(37858GD); act<CD2<Gal4, UAS-GFP/+*

Fig. S1C: *eyFLP/+; tub-Gal80*$^{ts}$*/UAS-Npl4*$^{RNAi}$ *(4673R-2); act<CD2<Gal4, UAS-GFP/+*

Fig. S1E: *eyFLP/+; tub-Gal80*$^{ts}$*/UAS-lgl*$^{RNAi}$*; act<CD2<Gal4, UAS-GFP/UAS-Ras*$^{V12}$

Fig. S1F: *eyFLP/+; tub-Gal80*$^{ts}$*/UAS-Ras*$^{V12}$*; act<CD2<Gal4, UAS-GFP/UAS-scrib*$^{RNAi}$

Fig. S1G: *eyFLP/+; tub-Gal80*$^{ts}$*/UAS-baz*$^{RNAi}$*; act<CD2<Gal4, UAS-GFP/UAS-Ras*$^{V12}$

Fig. S1H: *eyFLP/+; tub-Gal80*$^{ts}$*/UAS-Usp10*$^{RNAi}$ *(37858GD); act<CD2<Gal4, UAS-GFP/UAS-Ras*$^{V12}$

Fig. S1I: *eyFLP/+; tub-Gal80*$^{ts}$*/UAS-Npl4*$^{RNAi}$ *(4673R-2); act<CD2<Gal4, UAS-GFP/UAS-Ras*$^{V12}$

Fig. S1J: *eyFLP/+; tub-Gal80*$^{ts}$*/UAS- Ras*$^{V12}$*; act<CD2<Gal4, UAS-GFP/UAS-Usp47*$^{RNAi}$

Fig. S1K: *eyFLP/+; tub-Gal80*$^{ts}$*/UAS-not*$^{RNAi}$ *(45776GD); act<CD2<Gal4, UAS-GFP/UAS-Ras*$^{V12}$

Fig. S2F: *eyFLP/+; tub-Gal80*$^{ts}$*/UAS-P35; act<CD2<Gal4, UAS-GFP/+*

Fig. S2G: *eyFLP/+; tub-Gal80*$^{ts}$*/UAS-P35; act<CD2<Gal4, UAS-GFP/UAS-prp8*$^{RNAi}$ *(18567GD)*

Fig. S3O: *eyFLP/+; tub-Gal80*$^{ts}$*/UAS-rl; act<CD2<Gal4, UAS-GFP/UAS-prp8*$^{RNAi}$ *(18567GD)*

Fig. S4A: *eyFLP/UAS-EGFR*$^{\lambda Top4.1}$*; tub-Gal80*$^{ts}$*/+; act<CD2<Gal4, UAS-GFP/+*

Fig. S4B: *eyFLP/UAS-EGFR*$^{\lambda Top4.1}$*; tub-Gal80*$^{ts}$*/+; act<CD2<Gal4, UAS-GFP/UAS-prp8*$^{RNAi}$ *(18567GD)*

Fig. S4C: *eyFLP/+; tub-Gal80*$^{ts}$*/UAS-N*$^{ECD}$*; act<CD2<Gal4, UAS-GFP/MKRS*

Development • Supplementary information

Fig. S4D: *eyFLP/+; tub-Gal80*$^{ts}$*/UAS-N*$^{ECD}$*; act<CD2<Gal4, UAS-GFP/UAS-prp8*$^{RNAi}$ *(18567GD)*

Fig. S4E: *eyFLP/+; tub-Gal80*$^{ts}$*/UAS-mfap1*$^{RNAi}$ *(103419KK); act<CD2<Gal4, UAS-GFP/+*

Fig. S4F: *eyFLP/+; tub-Gal80*$^{ts}$*/UAS-mfap1*$^{RNAi}$ *(103419KK); act<CD2<Gal4, UAS-GFP/UAS-Ras*$^{V12}$

Fig. S4G: *eyFLP/+; tub-Gal80*$^{ts}$*/UAS-prp38*$^{RNAi}$ *(110282KK); act<CD2<Gal4, UAS-GFP/+*

Fig. S4H: *eyFLP/+; tub-Gal80*$^{ts}$*/UAS-prp38*$^{RNAi}$ *(110282KK); act<CD2<Gal4, UAS-GFP/UAS-Ras*$^{V12}$

Fig. S4I: *eyFLP/+; tub-Gal80*$^{ts}$*/+; act4<CD2<Gal4, UAS-GFP/UAS-bx42*$^{RNAi}$ *(34777 TRiP)*

Fig. S4J: *eyFLP/+; tub-Gal80*$^{ts}$*/UAS-Ras*$^{V12}$*; act4<CD2<Gal4, UAS-GFP/UAS-bx42*$^{RNAi}$ *(34777 TRiP)*

Fig. S7E: *w*; *ptc-Gal4, UAS-CD8-GFP/UAS-mfap1*$^{RNAi}$ *(103419KK)*

Fig. S7F: *w*; *ptc-Gal4, UAS-CD8-GFP/UAS-mfap1*$^{RNAi}$ *(103419KK); UAS-RET*$^{MEN2B\ (M955T)}$*/+*

Fig. S7G: *w; ptc-Gal4, UAS-CD8-GFP/UAS-prp38*$^{RNAi}$ *(110282KK)*

Fig. S7H: *w; ptc-Gal4, UAS-CD8-GFP/ UAS-prp38*$^{RNAi}$ *(110282KK); UAS-RET*$^{MEN2B\ (M955T)}$*/+*

Fig. S7I: *w*; *ptc-Gal4, UAS-CD8-GFP/+; UAS-bx42*$^{RNAi}$ *(34777 TRiP)/+*

Fig. S7J: *w*; *ptc-Gal4, UAS-CD8-GFP/+; UAS-bx42*$^{RNAi}$ *(34777 TRiP)/UAS-RET*$^{MEN2B\ (M955T)}$

Wing imaginal disc experiments were performed with *ptc-Gal4, UAS-CD8-GFP* (Nic Tapon). Crosses were performed at 25ºC until wandering L3 larvae were observed, collected and dissected.

Eye imaginal disc experiments were performed with *eyFLP*; *tub-Gal80*$^{ts}$; *act<CD2<Gal4*, *UAS-GFP*. Crosses were maintained at 18ºC for 120h before switching to 29ºC for induction of gene expression. Similar to wing imaginal disc experiments, wandering L3 larvae were collected and dissected after approximately 2-3 days at 29ºC.

Adult gut experiments were performed with *esg-Gal4*; *UAS-GFP*, *tub-Gal80*$^{ts}$. Crosses were maintained at 18ºC and flipped every 48 hours. Newly eclosed adult flies were collected and kept for 2 days at 18ºC before shifting to 29ºC for 2-7 days before dissection. Unless otherwise specified, all adult gut analyses were performed 7 days after transgene expression.

Development • Supplementary information

Mosaic analysis with a repressible cell marker (MARCM) clone experiments were performed with *y,w, hsFLP, UAS-GFP-nls; tub-Gal4, FRT42D tub-Gal80 / CyO*. Crosses were performed at 25ºC and 48h AEL larvae were heat-shocked for 1 hour. Wandering L3 larvae were collected, dissected and processed for immunofluorescence analysis.

Development • Supplementary information

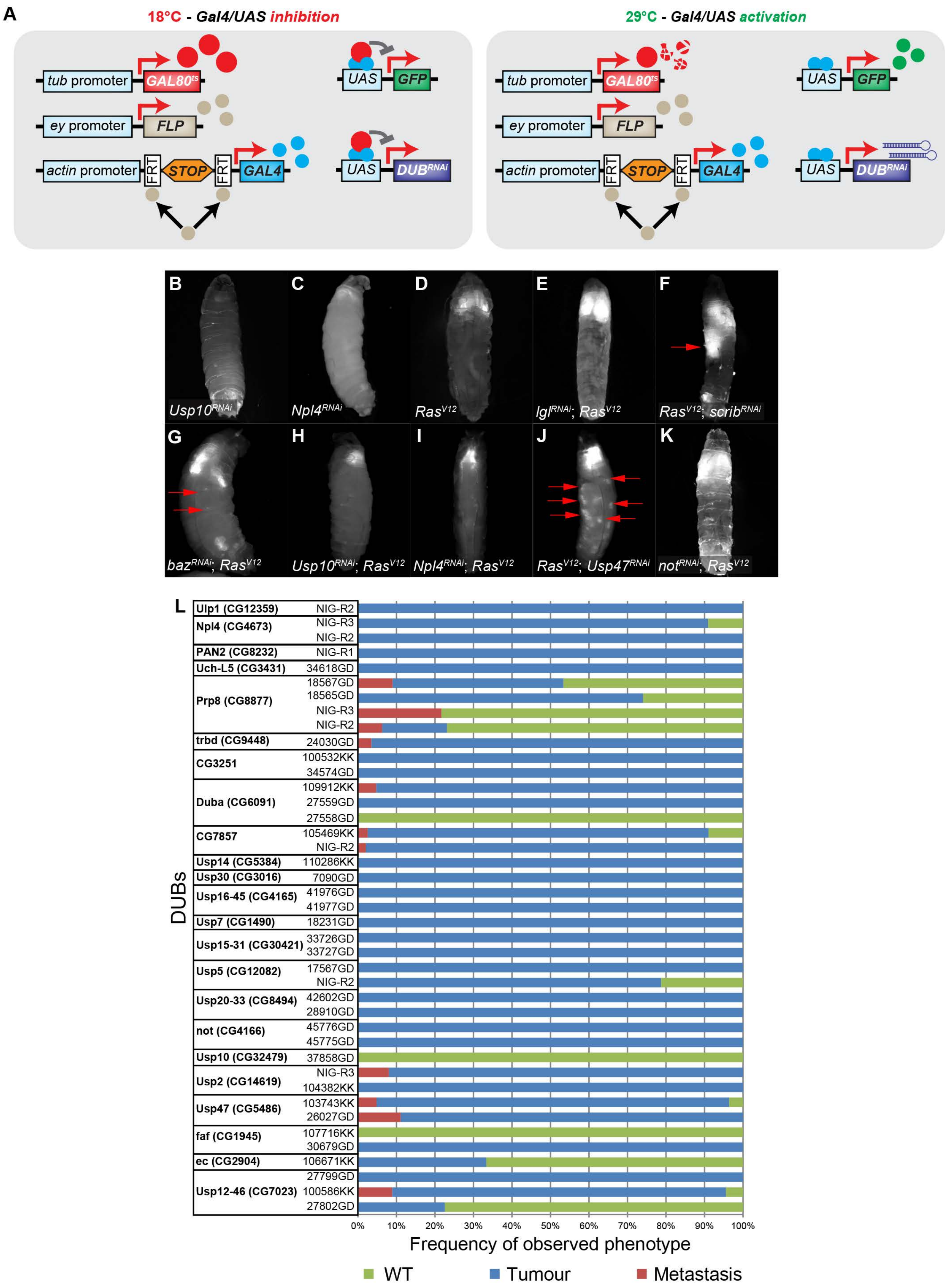
A
18°C - Gal4/UAS inhibition
29°C - Gal4/UAS activation
tub promoter
GAL80ts
ey promoter
FLP
actin promoter
FRT
STOP
FRT
GAL4
UAS
GFP
UAS
DUBRNAi
B
C
D
E
F
Usp10RNAi
Npl4RNAi
RasV12
lglRNAi; RasV12
RasV12; scribRNAi
G
H
I
J
K
bazRNAi; RasV12
Usp10RNAi; RasV12
Npl4RNAi; RasV12
RasV12; Usp47RNAi
notRNAi; RasV12
L
DUBs
Ulp1 (CG12359) NIG-R2
Npl4 (CG4673) NIG-R3
NIG-R2
PAN2 (CG8232) NIG-R1
Uch-L5 (CG3431) 34618GD
Prp8 (CG8877) 18567GD
18565GD
NIG-R3
NIG-R2
trbd (CG9448) 24030GD
CG3251 100532KK
34574GD
Duba (CG6091) 109912KK
27559GD
27558GD
CG7857 105469KK
NIG-R2
Usp14 (CG5384) 110286KK
Usp30 (CG3016) 7090GD
Usp16-45 (CG4165) 41976GD
41977GD
Usp7 (CG1490) 18231GD
Usp15-31 (CG30421) 33726GD
33727GD
Usp5 (CG12082) 17567GD
NIG-R2
Usp20-33 (CG8494) 42602GD
28910GD
not (CG4166) 45776GD
45775GD
Usp10 (CG32479) 37858GD
Usp2 (CG14619) NIG-R3
104382KK
Usp47 (CG5486) 103743KK
26027GD
faf (CG1945) 107716KK
30679GD
ec (CG2904) 106671KK
Usp12-46 (CG7023) 27799GD
100586KK
27802GD
0% 10% 20% 30% 40% 50% 60% 70% 80% 90% 100%
Frequency of observed phenotype
WT
Tumour
Metastasis

**Figure S1 – *In vivo* DUB$^{RNAi}$ screen in Ras$^{V12}$ background.**

**(A)** Schematic representation of genetic system controlling the expression of UAS transgenes in the developing eye imaginal disc. Temporal and spatial control of gene expression was achieved by combining *eyFLP* with an *act-Gal4* FLPout cassette and a *tub-Gal80$^{ts}$* element, such that expression is limited to *ey*-expressing regions upon incubation at 29ºC. **(B-K)** Images of third instar larvae showing distribution of GFP expression induced in the eye discs and optic lobes of the indicated genotypes. Note that (D) is the same representative *Ras$^{V12}$* larva as depicted in **Fig. 1B**. (B and C) depict other hits from RNAi screen, while (E-G) show screen validation experiments combining *Ras$^{V12}$* expression with RNAi-mediated depletion of polarity genes. The pattern of GFP distribution in whole larvae shows a variety of phenotypes of proliferation (E) and metastasis (F and G, red arrows). (H-K) are representative images of different phenotypes obtained with selected *DUB$^{RNAi}$* hits from the screen in combination with expression of *Ras$^{V12}$*. **(L)** Quantification of phenotype frequency for the indicated genotypes in combination with *Ras$^{V12}$* expression (n>60 larvae/genotype).

Development • Supplementary information

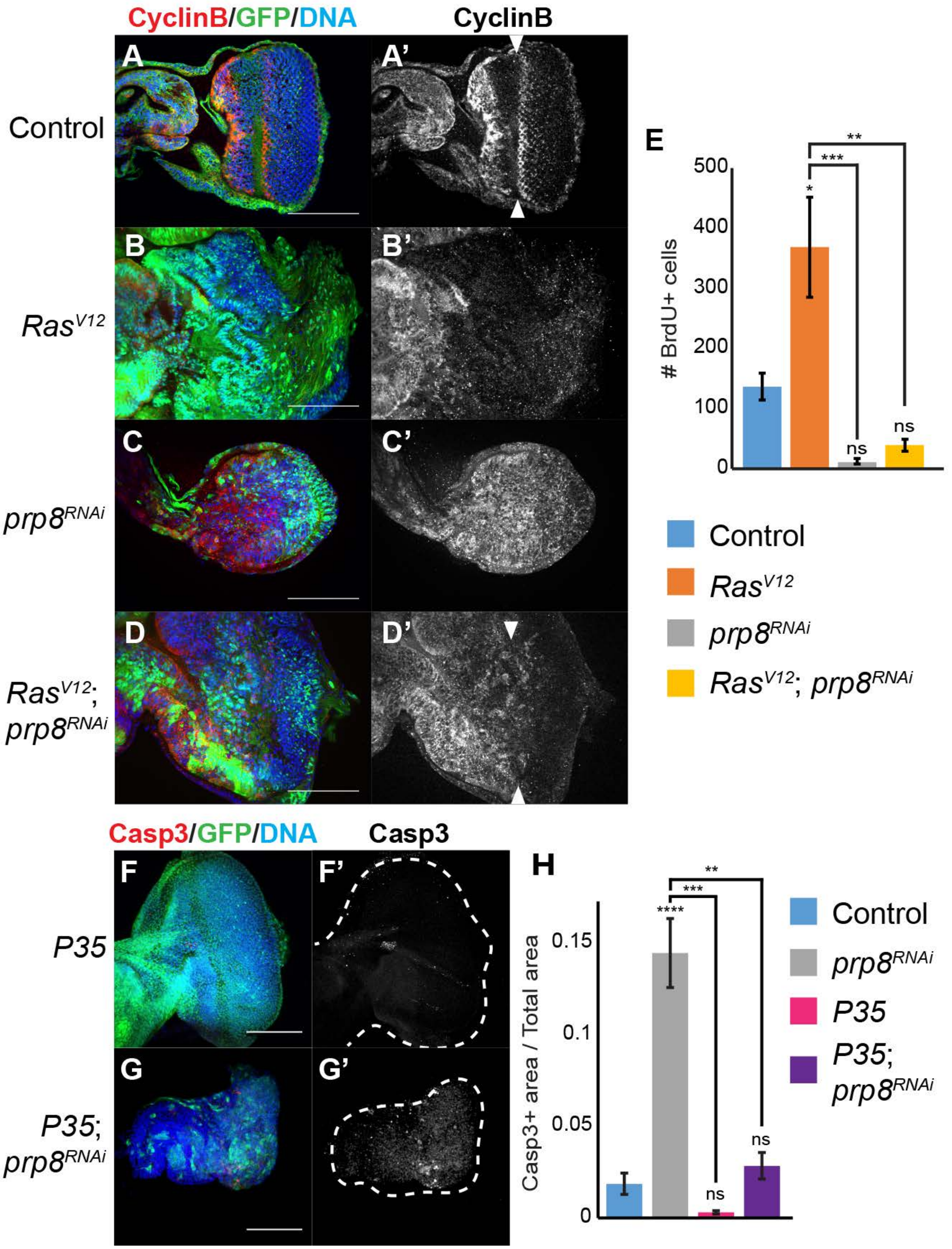
CyclinB/GFP/DNA
CyclinB
A
A'
Control
B
B'
RasV12
C
C'
prp8RNAi
D
D'
RasV12; prp8RNAi
E
500
400
300
200
100
0
# BrdU+ cells
*
***
**
ns
ns
Control
RasV12
prp8RNAi
RasV12; prp8RNAi
Casp3/GFP/DNA
Casp3
F
F'
P35
G
G'
P35; prp8RNAi
H
0.15
0.1
0.05
0
Casp3+ area / Total area
****
***
**
ns
ns
Control
prp8RNAi
P35
P35; prp8RNAi

**Figure S2 – Analysis of cell proliferation, cell cycle and apoptosis markers in *prp8*$^{RNAi}$-mediated eye disc hypoplasia.**

**(A-D)** Confocal micrographs of eye imaginal discs of the indicated genotypes, labelled with anti-Cyclin B (red in merged images (A-D) and gray in single-channel images (A'-D')), anti-GFP (green) and the DNA marker Hoechst (blue). **(E)** Quantification of number of BrdU-positive cells (BrdU+) in the indicated genotypes (n>4 discs/genotype). **(F,G)** Confocal micrographs of eye imaginal discs expressing the apoptosis inhibitor P35 alone (F) or in combination with *prp8*$^{RNAi}$ (G), labelled with anti-cleaved Caspase-3 (Dcp1) (red), anti-GFP (green) and the DNA marker Hoechst (blue). Eye discs expressing P35 alone displayed wild-type morphology (F), while expressing P35 in combination with *prp8*$^{RNAi}$ (G) was insufficient to rescue the hypoplasia phenotype (compare also with Fig. 1F and Fig. 2C). Note that eye discs where P35 was expressed in combination with *prp8*$^{RNAi}$ still showed a degree of cell death, which is likely to be caspase-independent (G'). **(H)** Quantification of the ratio between the area of Caspase-3-positive staining and the total eye disc area (n>4 discs/genotype). Note that the control and *prp8*$^{RNAi}$ data are the same as represented in **Fig. 2J**. Scale bars = 100 μm. Data are shown as mean ± SEM. * = p<0.05; ** = p<0.01; *** = p<0.001; **** = p<0.0001.

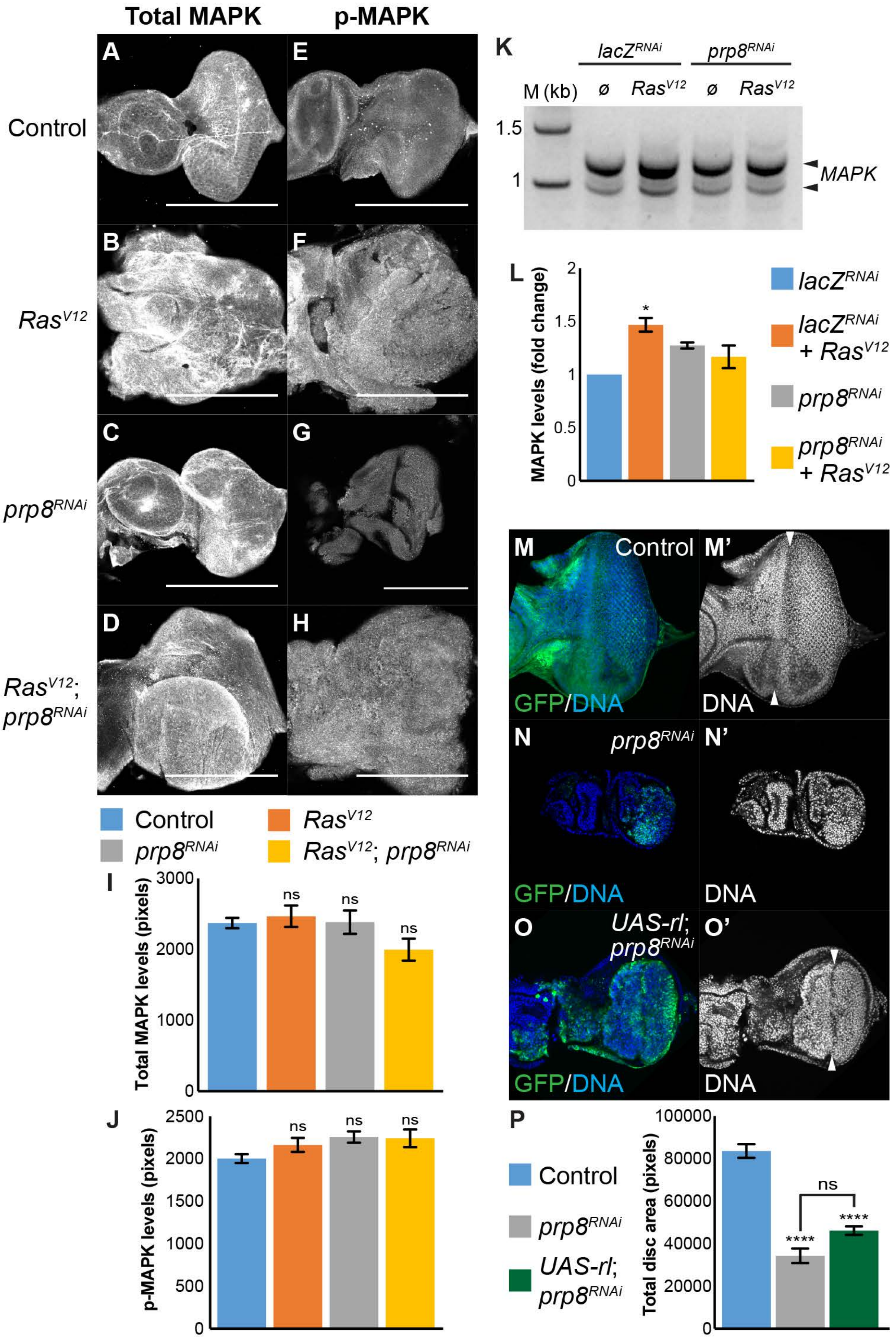


Development • Supplementary information

**Figure S3 – Effect of *prp8* depletion on MAPK signalling.**

**(A-H)** Confocal micrographs of eye imaginal discs of the indicated genotypes, stained for total MAPK (A-D) or phospho-MAPK (p-MAPK; E-H). **(I)** Quantification of total MAPK levels in eye discs (n>8 discs/genotype). **(J)** Quantification of p-MAPK levels in eye discs (n>5 discs/genotype). **(K)** RT-PCR analysis of MAPK splicing in S2 cells treated with control dsRNA ($lacZ^{RNAi}$) or dsRNA targeting *prp8* ($prp8^{RNAi}$) and transfected with a control plasmid (ø) or $Ras^{V12}$. **(L)** Quantification of fold change in MAPK expression levels from 2 independent RT-PCR experiments. **(M-O)** Confocal micrographs of eye imaginal discs of the indicated genotypes, stained for GFP (green) and the DNA marker Hoechst (blue (in M-O) or gray (in M'-O'). Arrowheads indicate position of morphogenetic furrow. **(P)** Quantification of eye disc area in the indicated genotypes (n>15 discs/genotype). Expression of MAPK (Rolled; rl) in eye discs depleted of *prp8* (O) is insufficient to suppress the $prp8^{RNAi}$ hypoplasia phenotype (N). Scale bars = 100 µm. Data are shown as mean ± SEM. * = p<0.05; **** = p<0.001. ns = non-significant.

Development • Supplementary information

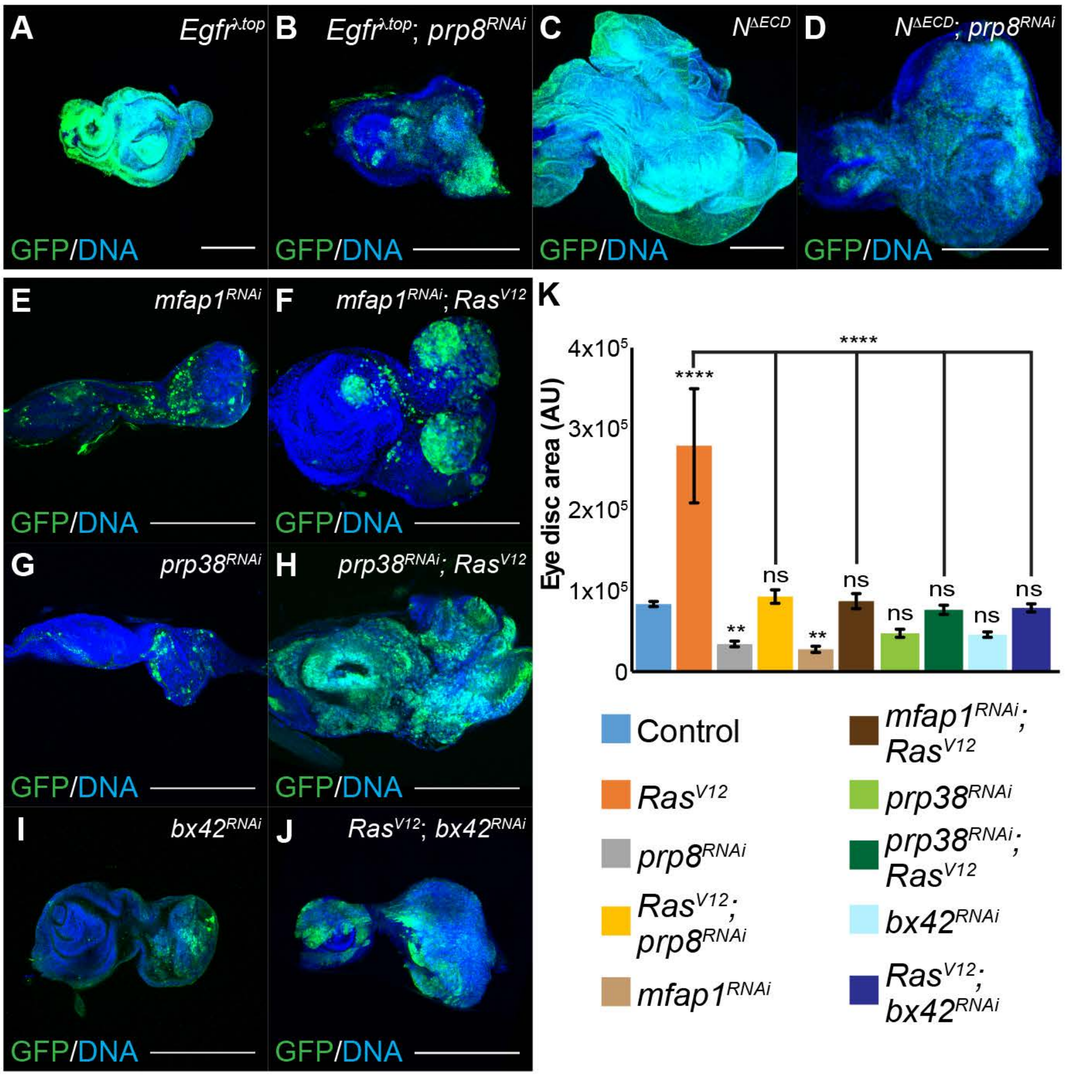


**Figure S4 – Effect of RNAi-mediated depletion of spliceosome components on Ras$^{V12}$-, EGFR- and Notch-induced hyperplastic growth.**

**(A-D)** Confocal micrographs of eye imaginal discs expressing an activated version of the EGF receptor (A; *Egfr$^{\lambda top}$*), or an activated version of Notch (C; *N$^{\Delta ECD}$*) alone or in combination with *prp8$^{RNAi}$* (B and D, respectively). Eye discs were labelled with anti-GFP (green) and the DNA-binding dye Hoechst (blue). Expression of either oncogene led to hyperplasia (A and C), which was significantly reduced in the presence of *prp8$^{RNAi}$*. **(E-J)** Confocal micrographs of eye imaginal discs of the indicated genotypes stained with anti-GFP (green) and the DNA marker Hoechst (blue). Note that depletion of spliceosome components in combination with *Ras$^{V12}$* expression suppresses the *Ras$^{V12}$* hyperplasia phenotype. **(K)** Quantification of eye disc area in the indicated genotypes (n>7 discs/genotype). Scale bar = 200 µm. Data are shown as mean ± SEM. ** = p<0.01; **** = p<0.001. ns = non-significant.

Development • Supplementary information

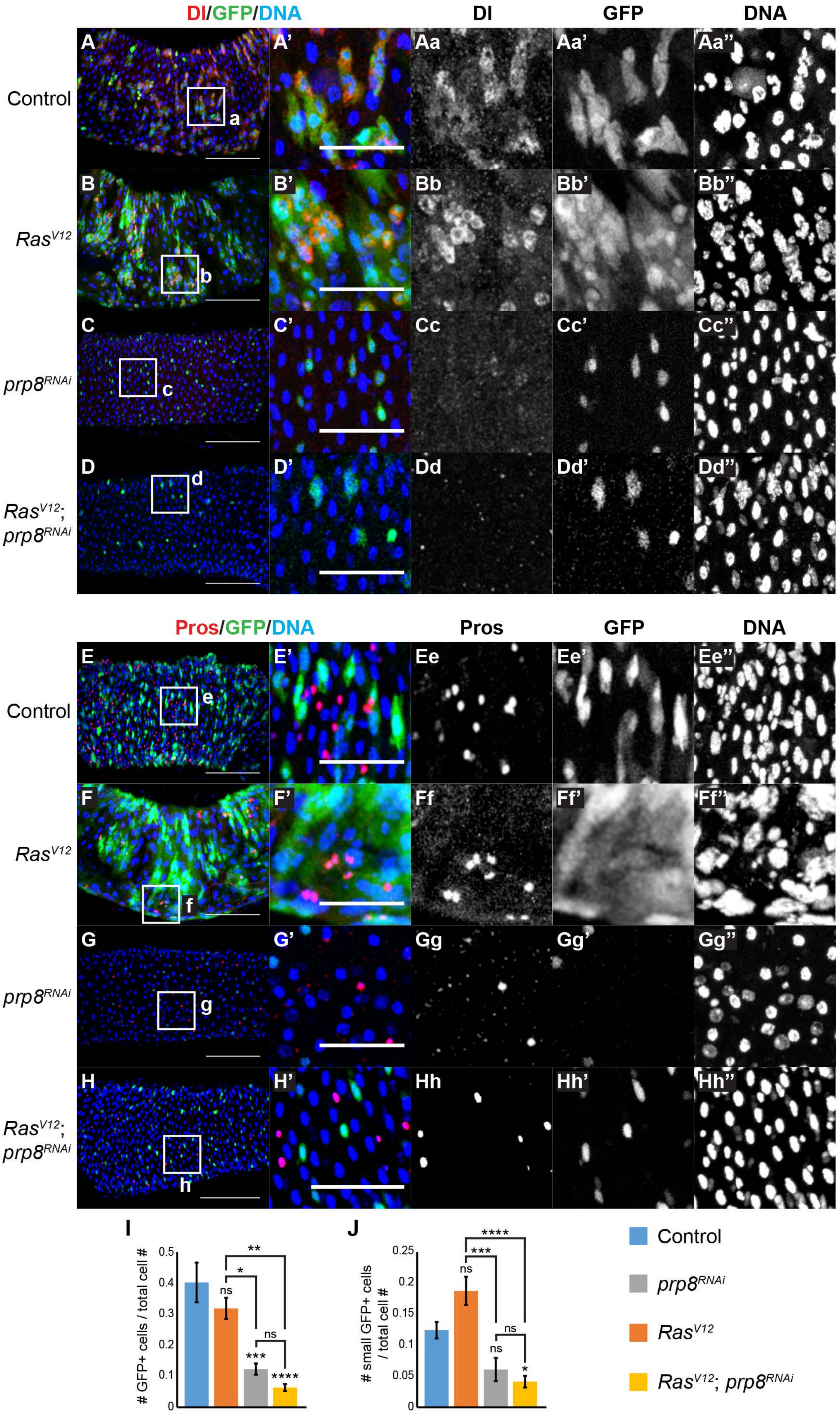


Development • Supplementary information

**Figure S5 – Prp8 regulates the number of stem cells and enteroendocrine cells in the adult gut.**

**(A-H)** Confocal micrographs of posterior midguts from adult flies of the indicated genotypes, stained for the Notch ligand Delta (Dl, red in A-D) or Prospero (Pros, red in E-H), GFP (green) and DNA (blue). **(a-h)** indicate regions of interest shown in magnified images **(A'-Hh'')**. (A'-H') show merged images, while (Aa-Hh'') depict individual channel images of the magnified region of interest. When compared with controls (A and E), *prp8*$^{RNAi}$ caused a reduction in the number of ISCs, which are both GFP- and Dl-positive (C) and a decrease in the number of enteroendocrine cells, which are marked by Pros expression (G). **(I)** Quantification of the ratio between the number of GPF-positive cells and the total number of cells in the posterior midgut, 7 days after induction (n>9 guts/genotype). **(J)** Quantification of the ratio between the number of small GFP-positive cells (ISCs and progenitors) and the total number of cells in the posterior midgut, 7 days after induction (n>9 guts/genotype). Scale bars = 100 µm (in whole midgut images A-F) and 40 µm (in magnified images). Data are shown as mean ± SEM. * = p<0.05; ** = p<0.01; *** = p<0.001; **** = p<0.0001.

Development • Supplementary information

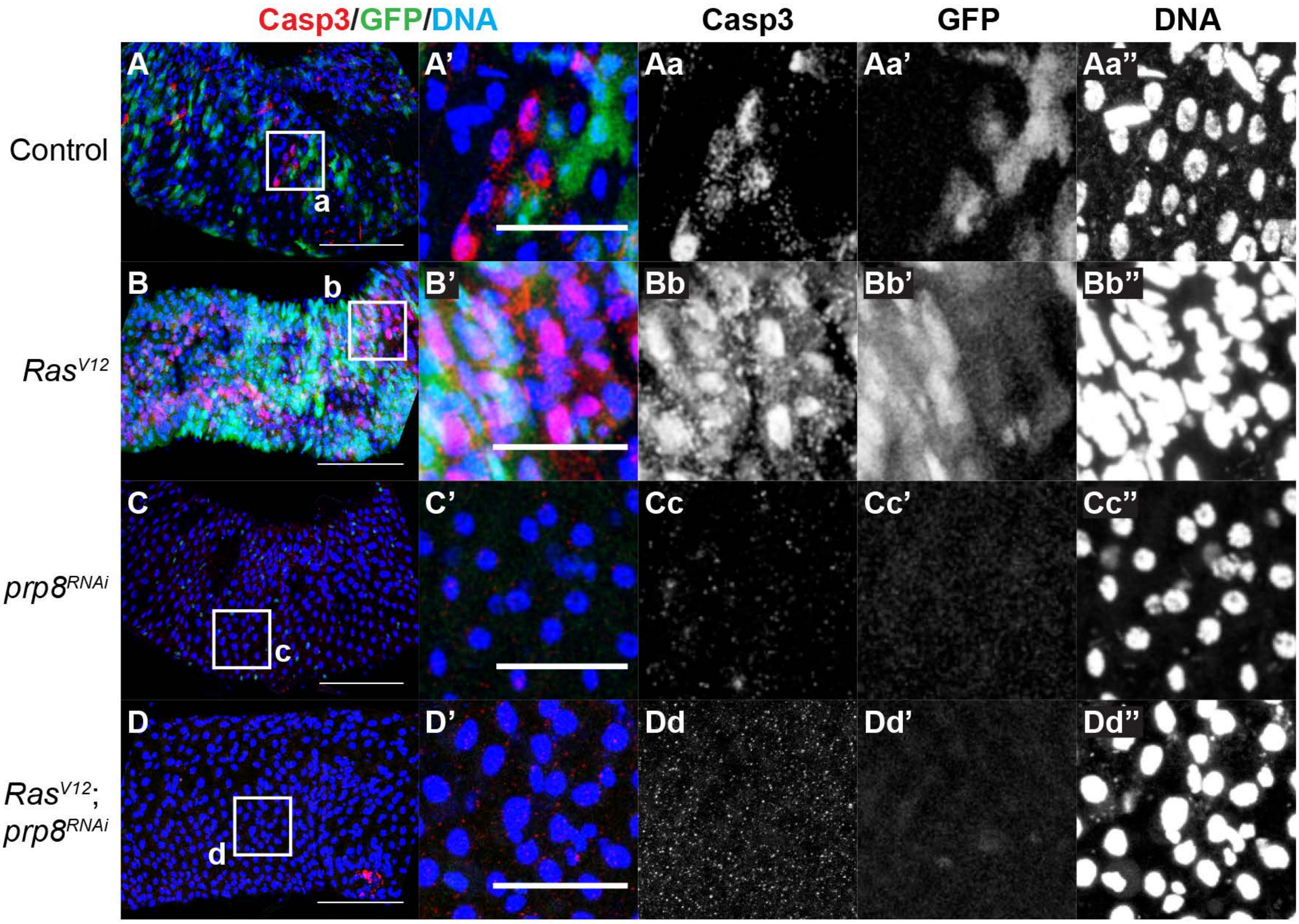


**Figure S6 – Prp8 loss is not associated with increased caspase activity in adult guts.**

**(A-D)** Confocal micrographs of posterior midguts from adult flies of the indicated genotypes, stained for activated Caspase-3 (Casp3, red), GFP (green) and DNA (blue) 4 days after transgene induction. **(a-h)** indicate regions of interest shown in magnified images **(A'-Dd'')**. (A'-D') show merged images, while (Aa-Dd'') depict individual channel images of the region of interest. *prp8*$^{RNAi}$ guts displayed reduced levels of activated Caspase-3 (C) when compared with both control (A) and *Ras*$^{V12}$-expressing flies (B). Scale bars = 100 µm (in whole midgut images A-F) and 40 µm (in magnified images).

Development • Supplementary information

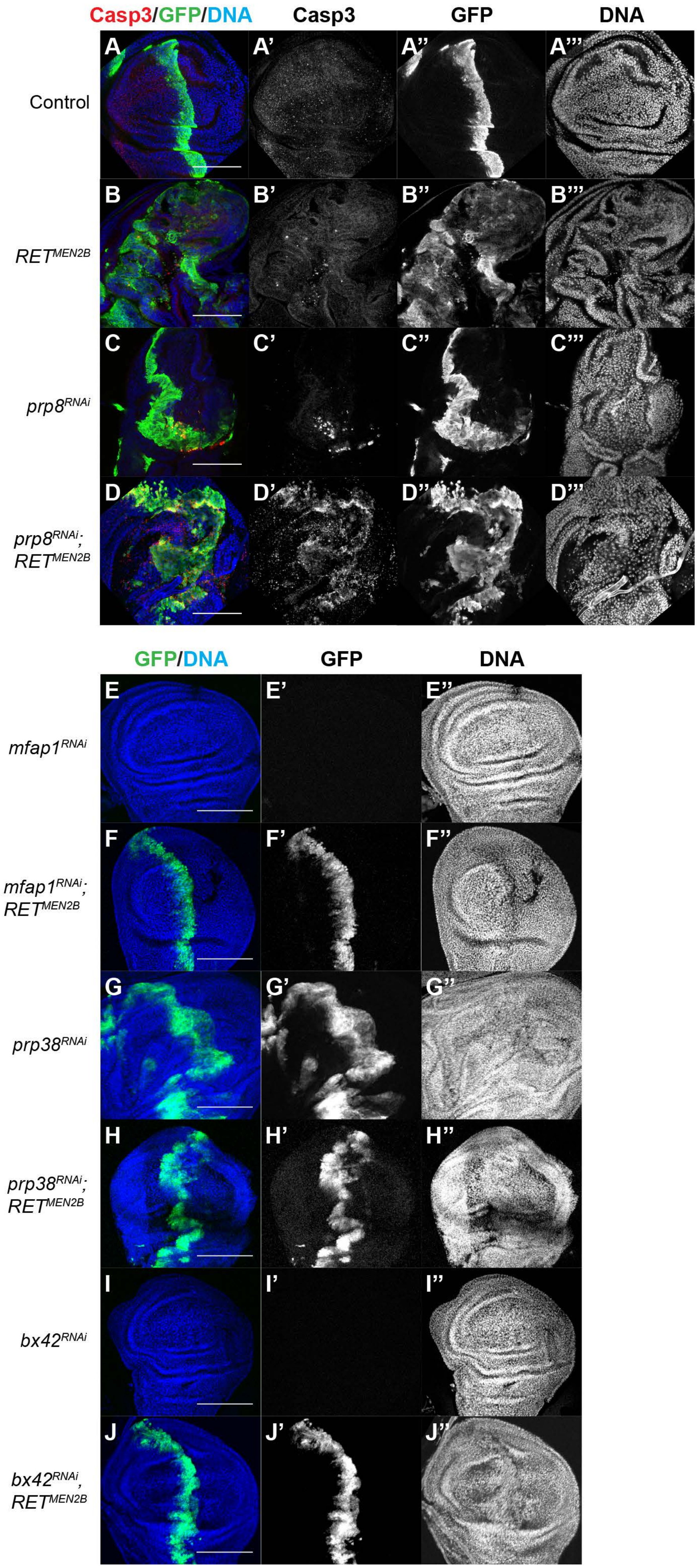

Casp3/GFP/DNA
Casp3
GFP
DNA
A
A'
A''
A'''
Control
B
B'
B''
B'''
RET^MEN2B
C
C'
C''
C'''
prp8^RNAi
D
D'
D''
D'''
prp8^RNAi;
RET^MEN2B
GFP/DNA
GFP
DNA
E
E'
E''
mfap1^RNAi
F
F'
F''
mfap1^RNAi;
RET^MEN2B
G
G'
G''
prp38^RNAi
H
H'
H''
prp38^RNAi;
RET^MEN2B
I
I'
I''
bx42^RNAi
J
J'
J''
bx42^RNAi;
RET^MEN2B

**Figure S7 – Caspase activity and effect of depletion of spliceosome components in wing disc tumour model**

**(A-D)** Confocal micrographs of wing imaginal discs from third instar larvae of the indicated genotypes, stained for activated Caspase-3 (Casp3, red), GFP (green) and DNA (blue). GFP expression marks the anterior/posterior boundary and the *ptc-Gal4*-expressing domain. $RET^{MEN2B}$ expression (B) and *prp8* depletion ($prp8^{RNAi}$; C) caused an increase in the GFP-positive area, when compared to controls (A) and, in the case of $prp8^{RNAi}$, this is associated with an increase in the levels of caspase activity (C'). Combining $RET^{MEN2B}$ and $prp8^{RNAi}$ led to enhancement of the $RET^{MEN2B}$ phenotype and the appearance of invasive cells outside the anterior/posterior boundary (D) and a marked increase in the levels of caspase activity (D'). **(E-J)** Confocal micrographs of wing imaginal discs from third instar larvae of the indicated genotypes, stained for GFP (green) and DNA (blue). Note that depleting components of the spliceosome resulted in the suppression of the $RET^{MEN2B}$ phenotype (compare F, H and J with B). Scale bars = 100 µm

Development • Supplementary information

**Table S1 – List of DUB RNAi lines used in the study**

| Type | Gene name | CG number | Mammalian Orthologue | RNAi lines |
|---|---|---|---|---|
| **DUB-USP** | Usp5 | CG12082 | USP5, USP13 (IsoT) | CG12082 VDRC 17567 GD<br>CG12082 NIG-FLY 12082-R2<br>CG12082 NIG-FLY 12082-R1 |
| **DUB-USP** | Usp2 | CG14619 | USP2, USP21 | CG14619 VDRC 104382<br>CG14619 NIG-FLY 14619-R1<br>CG14619 NIG-FLY 14619-R2<br>CG14619 NIG-FLY 14619-R3 |
| **DUB-USP** | Usp1 | CG15817 | USP1 | CG15817 VDRC 41604 GD<br>CG15817 VDRC 100992 KK<br>CG15817 VDRC 41605 GD |
| **DUB-USP** | Usp30 | CG3016 | USP30 | CG3016 VDRC 7090 GD |
| **DUB-USP** | Usp15-31 | CG30421 | USP31, USP43 | CG30421 VDRC 33727 GD<br>CG30421 VDRC 33726 GD<br>CG30421 VDRC 103553 KK |
| **DUB-USP** | Usp10 | CG32479 | USP10 | CG32479 VDRC 37858 GD<br>CG32479 VDRC 37859 GD |
| **DUB-USP** | Usp16-45 | CG4165 | USP16, USP45 | CG4165 VDRC 41977 GD<br>CG4165 VDRC 110286 KK<br>CG4165 VDRC 41976 GD |
| **DUB-USP** | Usp14 | CG5384 | USP14 | CG5384 VDRC 28647 GD<br>CG5384 VDRC 110227 KK<br>CG5384 VDRC 27405 GD |
| **DUB-USP** | puf | CG5794 | USP34 | CG5794 VDRC 27517 GD<br>CG5794 VDRC 106192 KK |
| **DUB-USP** | Usp8 | CG5798 | USP8 | CG5798 VDRC 107623 KK |
| **DUB-USP** | Usp12-46 | CG7023 | USP12, USP46 | CG7023 VDRC 27802 GD<br>CG7023 VDRC 100586 KK<br>CG7023 VDRC 27799 GD |
| **DUB-USP** | Usp32 | CG8334 | USP6, USP32 | CG8334 VDRC 18981 GD<br>CG8334 VDRC 18982 GD |
| **DUB-USP** | Usp20-33 | CG8494 | USP20, USP33 | CG8494 VDRC 42609 GD<br>CG8494 VDRC 28910 GD |
| **DUB-USP** | DUBAI | CG8830 | USP35, USP38 | CG8830 VDRC 28960 GD |
| **DUB-USP** | CYLD | CG5603 | CYLD | CG5603 VDRC 15340 GD<br>CG5603 VDRC 101414 KK |
| **DUB-USP** | ec | CG2904 | USP53, USP54 | CG2904 VDRC 106671 KK<br>CG2904 NIG-FLY 2904-R1 |
| **DUB-USP** | faf | CG1945 | USP9X, USP9Y | CG1945 VDRC 30679 GD<br>CG1945 VDRC 107716 KK |
| **DUB-USP** | not | CG4166 | USP22, USP27, USP51 | CG4166 VDRC 45775 GD<br>CG4166 VDRC 45776 GD |
| **DUB-USP** | Usp47 | CG5486 | USP47 | CG5486 VDRC 26027 GD<br>CG5486 VDRC 103743 KK |
| **DUB-USP** | scny | CG5505 | USP17, USP36, USP42 | CG5505 VDRC 105989 KK<br>CG5505 VDRC 11152 GD |
| **DUB-USP** | Usp7 | CG1490 | USP7 | CG1490 VDRC 18231 GD<br>CG1490 VDRC 110324 KK |
| **DUB-USP** | Usp39 | CG7288 | USP39 (SNUT2) | CG7288 NIG-FLY 7288-R1<br>CG7288 VDRC 47663 GD<br>CG7288 VDRC 47664 GD |
| **DUB-USP** | PAN2 | CG8232 | PAN2 (USP52) | CG8232 NIG-FLY 8232R-1 |
| **DUB-UCH** | Uch | CG4265 | UCHL1, UCHL3 | CG4265 VDRC 26468 GD<br>CG4265 VDRC 103614 KK |
| **DUB-UCH** | Uch-L5 | CG3431 | UCHL5 (UCH37) | CG3431 VDRC 34618 GD<br>CG3431 VDRC 103481 KK |
| **DUB-UCH** | Uch-L5R | CG1950 | UCHL5 (UCH37) | CG1950 NIG-FLY 1950R-1 |
| **DUB-UCH** | calypso | CG8445 | BAP1 | CG8445 VDRC 47743 GD<br>CG8445 VDRC 107757 KK |
| **DUB-MPN** | CG2224 | CG2224 | STAMBP, STAMBPL, AMSH | CG2224 VDRC 108622 KK<br>CG2224 NIG-FLY 2224-R1<br>CG2224 NIG-FLY 2224-R3 |
| **DUB-MPN** | CG4751 | CG4751 | MPND | CG4751 VDRC 45530 GD |
| **DUB-MPN** | CSN5 | CG14884 | COPS5 (JAB1) | CG14884 NIG-FLY 14884-R1<br>CG14884 NIG-FLY 14884-R3 |
| **DUB-MPN** | prp8 | CG8877 | PRP8 | CG8877 VDRC 18565 GD |

| | | | | |
|---|---|---|---|---|
| | | | | CG8877 VDRC 18567 GD<br>CG8877 NIG-FLY 8877-R2<br>CG8877 NIG-FLY 8877-R3 |
| **DUB-MPN** | Rpn11 | CG18174 | PSMD14 | CG18174 VDRC 19272 GD |
| **DUB-MPN** | Npl4 | CG4673 | NPLOC4 | CG4673 NIG-FLY 4673-R2<br>CG4673 NIG-FLY 4673-R3<br>CG4673 VDRC 109309 KK |
| **DUB-MPN** | eIF3f2 | CG8335 | second EIF3F | CG8335 VDRC 15507 GD<br>CG8335 VDRC 108169 KK |
| **DUB-MPN** | eIF3f1 | CG9769 | primary EIF3F | CG9769 VDRC 101465 KK |
| **DUB-MPN** | CSN6 | CG6932 | COPS6 | CG6932 VDRC 22308 GD<br>CG6932 VDRC 105385 KK |
| **DUB-MPN** | eIF3h | CG9124 | EIF3H | CG9124 VDRC 36087 GD |
| **DUB-MPN** | Rpn8 | CG3416 | PSMD7 | CG3416 VDRC 26183 GD<br>CG3416 VDRC 108573 KK |
| **DUB-OTU** | otu | CG12743 | OTUD4 (HIN1) | CG12743 VDRC 47431 GD<br>CG12743 VDRC 108845 KK |
| **DUB-OTU** | CG7857 | CG7857 | OTUD6A / OTUD6B | CG7857 NIG-FLY 7857-R2<br>CG7857 VDRC 105469 KK |
| **DUB-OTU** | CG3251 | CG3251 | OTUD4 (HIN1) | CG3251 VDRC 34573 GD<br>CG3251 VDRC 34574 GD<br>CG3251 VDRC 100532 KK |
| **DUB-OTU** | CG4968 | CG4968 | OTUB1 | CG4968 VDRC 21978 GD |
| **DUB-OTU** | CG4603 | CG4603 | YOD1 | CG4603 VDRC 21893 GD<br>CG4603 VDRC 21894 GD |
| **DUB-OTU** | Duba | CG6091 | OTUD5 | CG6091 VDRC 27558 GD<br>CG6091 VDRC 27559 GD<br>CG6091 VDRC 109912 KK |
| **DUB-OTU** | trbd | CG9448 | ZRANB1 (TRABID) | CG9448 VDRC 24030 GD |
| **DUB-Josph** | CG3781 | CG3781 | JOSD1 / JOSD2 | CG3781 VDRC 7113 GD<br>CG3781 VDRC 108379 KK |
| **ULP-SUMO** | Ulp1 | CG12359 | SENP1 / SENP2 | CG12359 VDRC 106625 KK<br>CG12359 NIG-FLY 12359-R2<br>CG12359 NIG-FLY 12359-R4 |
| **ULP-SUMO** | CG12717 | CG12717 | SENP6 / SENP7 | CG12717 VDRC 106239 KK |
| **ULP-SUMO** | CG1503 | CG1503 | SENP8 (DENP) | CG1503 VDRC 32349 GD<br>CG1503 VDRC 32350 GD<br>CG1503 VDRC 110486 KK |
| **ULP-SUMO** | Den1 | CG8493 | SENP8 (DENP) | CG8493 NIG-FLY 8493-R2<br>CG8493 VDRC 100591 KK<br>CG8493 VDRC 24110 GD |
| **ULP-SUMO** | velo | CG10107 | SENP6 / SENP7 | CG10107 VDRC 103524 KK |
| **ULP-NEDD8** | CG32110 | CG32110 | SENP1 / SENP2 | CG32110 VDRC 107634 KK<br>CG32110 VDRC 34064 GD<br>CG32110 VDRC 34062 GD |
| **DUB-MCPIP** | CG10889 | CG10889 | MCPIP1 (ZC3H12) | CG10889 NIG-FLY 10889-R2<br>CG10889 NIG-FLY 10889-R3<br>CG10889 VDRC 27330 GD |
| **DUB-MCPIP** | CG42360 | CG42360 | MCPIP2 | CG42360 VDRC 45755 GD<br>CG42360 VDRC 45755 GD |

Development • Supplementary information

**Table S2 – Summary of *in vivo* eye imaginal disc RNAi screen results for major hits**

| Gene name | RNAi lines | eyGal4 phenotype | Ras screen phenotype |
|---|---|---|---|
| Usp10 | CG32479 VDRC 37858 GD | eye disc hypoplasia | tumour reduction |
| | CG32479 VDRC 37859 GD | wt | ND |
| prp8 | CG8877 VDRC 18565 GD | eye disc hypoplasia | tumour reduction |
| | CG8877 VDRC 18567 GD | eye disc hypoplasia | tumour reduction/wt |
| | CG8877 NIG-FLY 8877-R2 | eye disc hypoplasia | tumour reduction/wt |
| | CG8877 NIG-FLY 8877-R3 | eye disc hypoplasia | tumour reduction/wt |
| Npl4 | CG4673 NIG-FLY 4673-R2 | wt | tumour |
| | CG4673 NIG-FLY 4673-R3 | wt | tumour |
| | CG4673 VDRC 109309 KK | eye disc hypoplasia | ND |

wt: wild-type phenotype; ND: not determined.
